# Association of a Risk Evaluation and Mitigation Strategy Program With Transmucosal Fentanyl Prescribing

**DOI:** 10.1001/jamanetworkopen.2019.1340

**Published:** 2019-03-29

**Authors:** William Fleischman, Doris Auth, Nilay D. Shah, Shantanu Agrawal, Joseph S. Ross

**Affiliations:** 1 Department of Patient Safety & Quality, Hackensack Meridian Health, Edison, New Jersey; 2 Centers for Medicare & Medicaid Services, Baltimore, Maryland; 3 US Food and Drug Administration, Silver Spring, Maryland; 4 Robert D. and Patricia E. Kern Center for the Science of Health Care Delivery, Mayo Clinic, Rochester, Minnesota; 5 Division of Health Care Policy and Research, Mayo Clinic, Rochester, Minnesota; 6 National Quality Forum, Washington, DC; 7 Center for Outcomes Research and Evaluation, Yale–New Haven Hospital, New Haven, Connecticut; 8 Department of Health Policy and Management, Yale School of Public Health, New Haven, Connecticut; 9 Section of General Internal Medicine, Department of Medicine, Yale School of Medicine, New Haven, Connecticut

## Abstract

**Importance:**

Transmucosal immediate-release fentanyl (TIRF) drugs are potent, rapid-acting opioids approved to treat breakthrough pain in patients with cancer who are tolerant to other around-the-clock opioid analgesics. In March 2012, a US Food and Drug Administration–approved Risk Evaluation and Mitigation Strategy (REMS) was implemented, mandating prescribers, distributors, pharmacies, and patients to enroll in the REMS to prescribe, dispense, or receive TIRF drugs.

**Objective:**

To evaluate the association of the TIRF-REMS Access Program with TIRF prescribing.

**Design, Setting, and Participants:**

Cohort study using an interrupted time series analysis of TIRF prescriptions to Medicare Part D beneficiaries nationwide from 2010 to 2014. Data were analyzed from August 2017 through July 2018.

**Main Outcomes and Measures:**

Prescribing of TIRF per 100 000 Medicare Part D beneficiaries, overall and stratified by cancer status; percentage of TIRF prescriptions for patients without cancer, overall and by brand; and percentage of TIRF prescriptions for patients without known opioid tolerance, defined as patients prescribed at least 60 morphine milligram equivalents per day, overall and by brand.

**Results:**

There were 99 601 TIRF prescriptions written by 8619 clinicians to 10 472 patients. Most of the patients (79%) were younger than 65 years (mean [SD] age, 56 [13] years), and most (67%) did not have cancer. Implementation of TIRF-REMS was associated with a 26.7% relative level decrease in TIRF prescribing (95% CI, −33.3% to −19.4%; *P* < .001) but was followed by 2.0% monthly increases in prescribing (95% CI, 1.3% to 2.7%; *P* < .001). Sensitivity analyses that accounted for overall opioid prescribing trends were consistent with these findings. Furthermore, there were no significant changes associated with REMS implementation in the level (0.47%; 95% CI, −5.36% to 4.69%; *P* = .85) or trend (0.16%; 95% CI, −0.06% to 0.37%; *P* = .15) of the percentage of prescriptions for patients without cancer. However, a sensitivity analysis that used a broader cancer definition found implementation was associated with a 7.2% (95% CI, −13.5% to −0.48%; *P* = .04) level decrease in the percentage of TIRF prescriptions for patients without cancer. Lastly, the TIRF-REMS was associated with a 22.5% level decline in the percentage of TIRF prescriptions for patients without known opioid tolerance (95% CI, −36.1% to −5.95%; *P* = .01) followed by 1.98% monthly decreases (95% CI, −3.19% to −0.80%; *P* = .001).

**Conclusions and Relevance:**

Implementation of the TIRF-REMS Access Program, a restrictive drug distribution program, was associated with a temporary reduction in the rate of TIRF prescribing to Medicare Part D beneficiaries, and with a sustained decrease in the percentage of TIRF prescriptions for patients without known opioid tolerance. Implementation may have also been associated with a temporary decrease in the percentage of TIRF prescriptions for patients without cancer.

## Key Points

**Question:**

Was the implementation of the Transmucosal Immediate-Release Fentanyl (TIRF)–Risk Evaluation and Mitigation Strategy (REMS) associated with changes in prescribing of TIRF medications?

**Findings:**

In this cohort study using interrupted times series analysis, implementation of TIRF-REMS was associated with a temporary reduction in the rate of overall TIRF prescribing to Medicare Part D beneficiaries and with a sustained decrease in the percentage of TIRF prescribed to patients without known opioid tolerance. The TIRF-REMS program may have also been associated with a temporary decrease in the percentage of TIRF prescribed to patients without cancer.

**Meaning:**

Mandatory, restrictive drug distribution programs, such as the TIRF-REMS, may be associated with changes in opioid prescribing, although the changes may be temporary.

## Teaser

This cohort study investigates the association between implementation of a risk evaluation and mitigation strategy program with transmucosal fentanyl prescribing for Medicare Part D beneficiaries.

## Introduction

Drug overdoses were the leading cause of accidental death in the United States in 2016.^1^ The opioid overdose epidemic has been attributed, in part, to a rapid increase in opioid analgesic prescribing in the period between 1990 and 2010.^2^ After peaking in 2012, US opioid analgesic prescribing rates have decreased slightly,^3^ although the amount prescribed in 2015 was still approximately 3 times as high as in 1999.^4^

Transmucosal immediate-release fentanyl (TIRF) products are potent, rapid-acting opioid analgesics only approved for use for breakthrough pain in patients with cancer who are already receiving and who are known to be tolerant to around-the-clock opioid analgesics. Reports have, however, suggested there is substantial off-label prescribing of TIRF products.^5,6^ To address their growing use and potential harms, the US Food and Drug Administration (FDA) has required TIRF manufacturers to institute programs to restrict prescribing and promote appropriate opioid prescribing and safe use of opioid analgesics, including Risk Evaluation and Mitigation Strategies (REMS). A REMS is a drug safety program that the FDA can require for certain medications with serious safety concerns to help ensure the benefits of the medication outweigh its risks.

In December 2011, the FDA approved the TIRF-REMS Access Program, mandating prescribers, distributors, pharmacies, and patients to enroll in the program before they can prescribe, distribute, dispense, or use TIRF products.^7^ The TIRF-REMS Access Program was fully implemented in March 2012 and is a shared system REMS that includes all TIRF products. Under the TIRF-REMS, prescribers and pharmacists are required to complete an educational program, patients must be enrolled and are required to sign an agreement attesting to their understanding of the risks and safe use of TIRFs, and distributors are required to agree to ship TIRF products only to enrolled pharmacies. Education for prescribers and pharmacists focuses on key safety information for minimizing the drug’s risks and on the safe and appropriate use of these medications. The association of TIRF-REMS with TIRF prescribing has, to our knowledge, not been previously described.

We sought to evaluate the association of the TIRF-REMS Access Program with TIRF prescribing using interrupted time series analyses. We used 2010 through 2014 Medicare Part D claims to evaluate TIRF prescribing overall, among patients without cancer, and among patients without known opioid tolerance. We examined whether implementation of the TIRF-REMS was associated with changes in prescribing.

## Methods

### Data Sources

The Yale University institutional review board exempted this study from review. The study followed the Strengthening the Reporting of Observational Studies in Epidemiology (STROBE) reporting guideline. The data were obtained in April 2017 and analyzed from August 2017 through July 2018. We assessed TIRF prescribing using prescription claims data submitted to Medicare Part D for 2010 through 2014. Medicare Part D provided drug coverage for 29.5 million beneficiaries in 2010, including adults aged 65 years or older, as well as disabled adults and those with end-stage renal disease younger than 65 years. Part D grew to cover 40 million beneficiaries in 2014, which represented 70% of eligible Medicare beneficiaries.^8^ We linked the pharmacy claims to beneficiary-level administrative claims data over the study period using beneficiary identifiers. Of note, Part D claims represent dispensed prescriptions; a clinician’s act of prescribing does not by itself generate a claim.

### Opioid and TIRF Prescriptions

Using a 100% Part D file, we identified all prescription claims for opioids (eTable 1 in the Supplement) using the opioid class identifier of the Medi-Span Master Drug Database classification system, which is used to categorize drug claims (eAppendix 1 in the Supplement). We then identified claims for TIRF products (eTable 2 in the Supplement) using the drug component (fentanyl) and route (oral, intranasal, sublingual, or buccal). We excluded prescriptions missing the route and prescriptions for injectable medications, as detailed in eAppendix 2 in the Supplement.

### Outcomes

Our main outcome measures were TIRF prescription fills per 100 000 Medicare Part D beneficiaries, overall and stratified by cancer status, drug brand, and age group; the percentage of TIRF prescriptions for patients without cancer, overall and by brand; and the percentage of TIRF prescriptions for patients without known opioid tolerance, overall and by brand. As a secondary outcome, we characterized the number of unique prescribers before and after program implementation.

To determine TIRF prescribing per 100 000 Medicare Part D beneficiaries, we calculated aggregate monthly opioid and TIRF prescribing rates per 100 000 Part D beneficiaries, accounting for the monthly variation in covered beneficiaries using Part D monthly enrollment data.^9^ We excluded prescriptions for sublingual fentanyl tablets marketed as Abstral, fentanyl nasal spray marketed as Lazanda, and fentanyl buccal film marketed as Onsolis (0.96%, 0.49%, and 0.24% of all TIRF prescriptions, respectively), as these drugs had separate REMS implemented prior to the classwide TIRF-REMS implementation. We identified unique prescribers using national provider identifiers or US Drug Enforcement Agency registration numbers included in the prescription data. Of note, the only brands for which there were prescriptions during each month of the study period to allow for brand-level analysis were Actiq and Fentora.

To calculate the percentage of TIRF prescriptions for patients without cancer, we first needed to differentiate patients with and without cancer. We categorized prescriptions filled by beneficiaries who did not have a cancer diagnosis associated with any visit during the calendar year the prescription was filled as TIRF prescriptions for patients without cancer. In contrast, prescriptions filled by beneficiaries with a cancer diagnosis associated with any visit during the same calendar year were categorized as prescriptions for patients with cancer. We also performed sensitivity analysis for this primary outcome measure using a broader cancer definition, defined as beneficiaries who had a cancer diagnosis associated with any visit during any year of the study period. To determine cancer diagnoses, we used 4 hierarchical condition category indicators^10^ that encompass all cancer diagnoses (indicators 7-10). These hierarchical condition category indicators are determined annually for all Medicare beneficiaries using inpatient and outpatient claims and are part of a model used by CMS to risk adjust Medicare beneficiaries and determine payments for Medicare Advantage insurers. For each month, we calculated the percentage of TIRF prescriptions for patients without cancer, overall and by brand. We again excluded prescriptions for Abstral, Lazanda, and Onsolis, and brand-level analyses were only conducted for Actiq and Fentora.

To calculate the percentage of TIRF prescriptions for patients without known opioid tolerance, we first needed to define opioid tolerance. To do this, we used the definition of opioid tolerance in the approved TIRF-REMS education program,^11^ which defines it as those patients receiving opioids averaging 60 morphine milligram equivalents (MME) per day for at least 7 days, or who have been receiving 30 mg of oxycodone or 8 mg of hydromorphone daily for at least 7 days. We calculated the total MME for each prescription using Centers for Disease Control and Prevention published conversion factors (eTable 3 in the Supplement).^12^ We then calculated the average daily prescribed MME for each patient using all opioid prescriptions filled in the 7-, 14-, 30-, 60-, and 90-day periods prior to the patient’s initial TIRF prescription, including prescriptions for Abstral, Lazanda, and Onsolis. Patients whose daily prescribed average was less than 60 MME in all of the lookback periods and whose average daily dose of oxycodone or hydromorphone was less than 30 mg and 8 mg, respectively, were considered patients without known opioid tolerance. We then calculated the monthly percentage of initial TIRF prescriptions to patients without known opioid tolerance, overall and by brand. Of note, for these analyses, the only brand for which there were prescriptions during each month of the study period to allow for brand-level analysis was Fentora.

### Statistical Analysis

After descriptive analysis, we performed interrupted time series analyses using segmented ordinary least squares regression^13^ with robust Newey-West errors to account for autocorrelation and heteroskedasticity.^14,15^ Interrupted time series analysis is best used to analyze data collected over time and at regular intervals.^16^ This method of analysis calculates independent tests of the level (the intercept) and trend (slope) before and after an interruption, or intervention, and then evaluates for differences between the 2 levels and slopes. This allows the preintervention level and trend to serve as a predictor of the counterfactual, ie, what would have been without the intervention.^17^ Additional details on the models used are included in eAppendix 3 in the Supplement.

We assessed whether REMS implementation in March 2012 was associated with changes in either the level or trend for the 3 main outcome measures. Changes in level would be expected around intervention implementation, while trend tracks outcomes in the preintervention and postintervention periods. We specified a level change model,^18^ expecting the strict implementation and mandatory requirements and controls of the TIRF-REMS program to have an immediate impact on TIRF prescribing overall, as well as on prescribing to patients without cancer and to patients without known opioid tolerance.

Analyses for the main outcome measures were performed using single-group time series analysis. Single-group time series models assume that the preintervention trend captures all unmeasured time-varying confounders.^14,17^ To account for potential unmeasured confounders that could have affected all opioid prescribing, such as a growing national awareness of opioid harms, we performed sensitivity analyses using all-opioid prescribing as a control. For these analyses we used prescriptions for any product containing opioids during the study period, and also repeated the analysis excluding prescriptions for buprenorphine-containing products, as an increase in buprenorphine prescribing for opioid use disorder treatment could mask a decrease in overall opioid prescribing, as detailed in eAppendix 4 in the Supplement. We used multiple-group time series models to look for differences between all-opioid prescribing and TIRF prescribing. We also used 2-group models to assess for differences in outcome measures between brands, using generic prescriptions as a control.

All models were adjusted to account for autocorrelation, seasonal variation, and the number of days in the month, as detailed in eAppendix 5 in the Supplement. Statistical significance was considered as 2-sided *P* < .05. We report adjusted relative percentage changes in the text, with adjusted absolute level and slope changes in the Table, as detailed in eAppendix 6 in the Supplement. Analysis was performed using Stata statistical software version 14.1 (StataCorp LLC^14^).

**Table.** Interrupted Time Series Regression Analysis for the Primary Outcomes^a^

Outcome	Baseline Trend (95% CI)	Postintervention (95% CI)	
		Level Change	Trend
Per 100 000 Part D beneficiaries^b^			
All opioid prescriptions	11.2 (−8.3 to 30.8)	39.5 (−299.2 to 378.3)	−64.9 (−92.8 to −37.0)
TIRF prescriptions	−0.06 (−0.09 to −0.03)	−1.25 (−1.70 to −0.80)	0.10 (0.06 to 0.13)
% Of TIRF prescriptions for patients^c^			
Without cancer	−0.22 (−0.34 to −0.10)	−0.21 (−3.79 to 3.36)	0.12 (−0.03 to 0.28)
Without known opioid tolerance	0.16 (−0.11 to 0.43)	−6.43 (−10.00 to −1.96)	−0.46 (−0.74 to −0.17)

Abbreviation: TIRF, transmucosal immediate-release fentanyl.

^a^ Primary outcomes include monthly prescribing for all opioids and transmucosal immediate-release fentanyl drugs, the percentage of TIRF prescriptions for beneficiaries without cancer, and the percentage of TIRF prescriptions for patients without known opioid tolerance from 2010 to 2014.

^b^ The table presents absolute values, with relative percentage changes in the text.

^c^ The table presents absolute percentage changes, with relative percentage changes in the text. Results are adjusted for seasonal trends and autocorrelation.

## Results

### Overall Rates of TIRF Use

**Figure 1.**
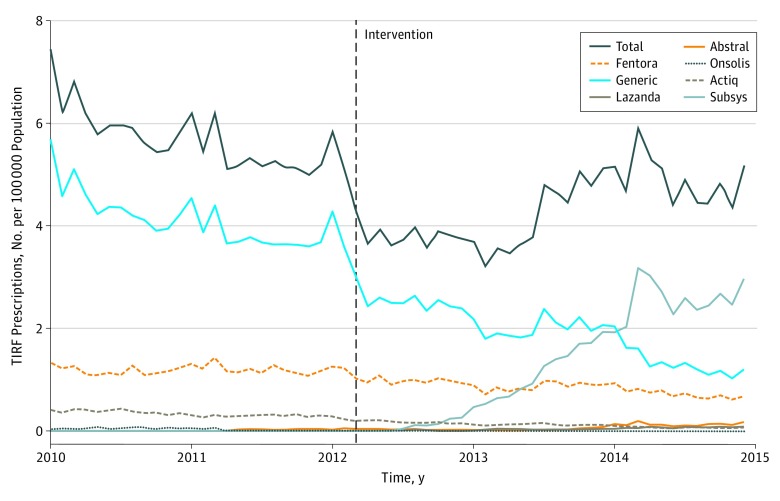
Transmucosal Immediate-Release Fentanyl (TIRF) Prescriptions per 100 000 Part D Participants 2010-2014, by Drug Brand

During calendar years 2010 through 2014, there were 99 601 TIRF prescriptions filled, written by 8619 clinicians to 10 472 patients. For context, over the same period there were 372 023 319 opioid prescriptions filled, written by 2 001 523 clinicians to 27 409 105 patients. Prescriptions for TIRF products represented 0.03% of all opioid prescriptions. The mean (SD) age of patients who filled TIRF prescriptions was 56 (13) years. More than three-quarters (79%) of TIRF prescriptions were for patients younger than 65 years. The TIRF products, including brand names, prescribed during the study period are listed in eTable 3 in the Supplement and prescribing trends by brand are shown in Figure 1.

**Figure 2.**
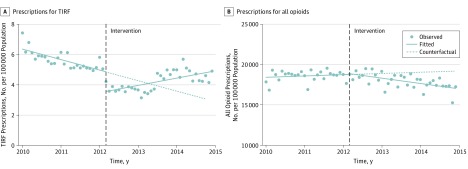
Monthly Prescriptions per 100 000 Part D Participants for Transmucosal Immediate-Release Fentanyl (TIRF) Drugs and All Opioids, 2010-2014 Points represent the raw data; solid lines represent the unadjusted, best-fit slope; and the dotted line represents the counterfactual, the predicted line without intervention. Adjusted models are included in eFigure 1 in the Supplement.

In the period prior to TIRF-REMS program implementation, TIRF prescribing decreased by 1.0% per month (95% CI, −1.5% to −0.6%; *P* < .001) (Table, Figure 2A; eFigure 1A in the Supplement; for clarity, figures in the article show unadjusted models, whereas adjusted models are shown in the Supplement). The TIRF-REMS implementation was associated with a 26.7% level decline in TIRF prescribing (95% CI, −33.3% to −19.4%; *P* < .001) but was followed by a change in the trend from 1.0% monthly decreases in prescribing to 2.0% monthly increases (95% CI, 1.3%-2.7%; *P* < .001). This trend change was driven by prescribing increases that began approximately a year after TIRF-REMS implementation.

In contrast to TIRFs, the rate of all-opioid prescribing did not vary significantly prior to the TIRF-REMS program implementation (slope = 0.06%, 95% CI, −0.04% to 0.20%; *P* = .24) (Figure 2B; eFigure 1B in the Supplement). In addition, there was no significant level change in all-opioid prescribing associated with TIRF-REMS implementation (level change, 0.26%; 95% CI, −1.7% to 2.2%; *P* = .79), with 0.36% monthly declines during the postimplementation period (95% CI, −0.52% to −0.21%; *P* < .001). A sensitivity analysis using a 2-group interrupted time series model with all-opioid prescribing as a control showed similar results (eFigure 2 in the Supplement); repeat analyses that excluded buprenorphine from the all-opioid control group showed similar results.

Supplementary analyses showed no significant difference in prescribing patterns by age group associated with TIRF-REMS implementation (eFigure 3 in the Supplement). Similarly, there were no significant brand-related prescribing differences associated with TIRF-REMS implementation (eFigure 4 in the Supplement). However, post hoc, supplementary analyses that exclude Subsys prescriptions are shown in eFigure 5 in the Supplement.

Notably, we observed a discordance between prescribing levels and number of prescribers. There was an initial decrease in TIRF prescribing accompanied by a similarly steep decrease in the number of unique TIRF prescribers. Subsequently we observed a return to baseline prescribing levels, despite a sustained decrease in the number of prescribers (eFigure 6 in the Supplement).

### TIRF Prescribing to Patients With and Without Cancer

**Figure 3.**
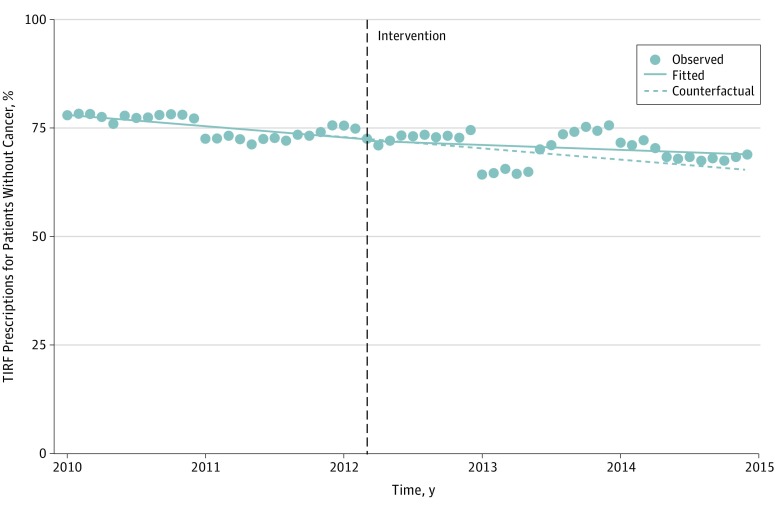
Monthly Percentage of Transmucosal Immediate-Release Fentanyl (TIRF) Prescriptions to Patients Without Cancer, 2010-2014 Points represent the raw data; solid lines represent the unadjusted, best-fit slope; and the dotted line represents the counterfactual, the predicted line without intervention. Adjusted and sensitivity models are included in eFigures 7-10 in the Supplement.

Most of the patients who filled TIRF prescriptions (67%) did not have a diagnosis of cancer associated with their claims during the calendar year of the filled prescription, and most prescriptions (72%) were for patients without cancer. There was a 0.29% monthly decline in the percentage of TIRF prescriptions for patients without cancer during the pre–TIRF-REMS period (95% CI, −0.45% to −0.14%; *P* = .001) (Figure 3). There were no significant changes associated with REMS implementation in the level (−0.47%; 95% CI, −5.36% to 4.69%; *P* = .85) or trend (0.16%; 95% CI, −0.06% to 0.37%; *P* = .15) of the percentage of TIRF prescriptions for patients without cancer. Estimated declines in the level of TIRF prescribing after TIRF-REMS implementation were similar for patients without cancer (−27.0%; 95% CI, −36.1% to −16.6; *P* < .001) to those with cancer diagnoses during the calendar year of the prescription (−26.9%; 95% CI, −34.0% to −19.0%; *P* < .001) (eFigure 7 and eFigure 8 in the Supplement).

Sensitivity analyses using a broader cancer definition (a claim with a cancer diagnosis during any year of the study) found a similar pre-REMS trend but also found REMS implementation was associated with a 7.2% level decrease in the percentage of TIRF prescriptions for patients without cancer (95% CI, −13.5% to −0.48%; *P* = .04) and with a change in the prescribing trend, from monthly decreases of 0.29% (95% CI, −0.49% to −0.10%; *P* = .003) to monthly 0.63% increases (95% CI, 0.28%-0.98%; *P* = .001) (eFigure 9 in the Supplement). As with the findings for the overall TIRF prescribing rate, in this sensitivity analysis the increase in prescribing began approximately a year after TIRF-REMS implementation.

Sensitivity analysis comparing brand to generic prescriptions found no consistent differences in prescriptions for patients without cancer for Actiq prescriptions compared with generic, while Fentora had an associated 8.3% level decline in filled prescriptions (95% CI, −14.8% to −1.27%, *P* = .02) (eFigure 10 in the Supplement). The monthly percentage of TIRF prescriptions for patients without cancer by brand are shown in eFigure 11 in the Supplement.

### Prescribing to Patients Without Known Opioid Tolerance

**Figure 4.**
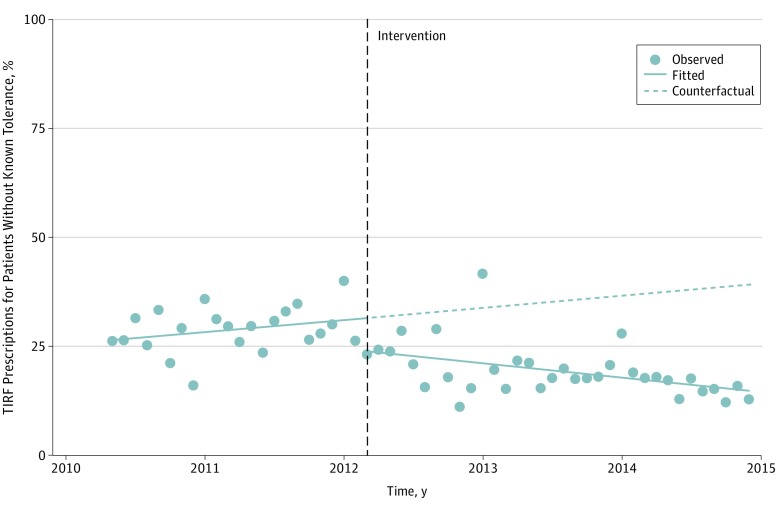
Monthly Percentage of Initial Transmucosal Immediate-Release Fentanyl (TIRF) Prescriptions to Patients Without Known Opioid Tolerance, 2010-2014 Points represent the raw data; solid lines represent the unadjusted, best-fit slope; and the dotted line represents the counterfactual, the predicted line without intervention. For this analysis we excluded the first 3 months of 2010, as we used an up to 90-day lookback period to establish patients’ prescribing history and known opioid tolerance. Adjusted and sensitivity models are included in eFigure 12 and eFigure 14 in the Supplement.

Prior to TIRF-REMS implementation, a mean of 30% of initial TIRF prescriptions were for patients without known opioid tolerance as defined by the REMS guidelines, which was nonsignificantly increasing monthly (0.60%, 95% CI, −0.46% to 0.17%; *P* = .26) (Figure 4; eFigure 12 in the Supplement). Implementation of TIRF-REMS was associated with a 22.5% relative level decline in the percentage of TIRF prescriptions filled by patients without known opioid tolerance (95% CI, −36.1% to −5.95%; *P* = .01) followed by 1.98% monthly decreases (95% CI, −3.19% to −0.80%; *P* = .001). The decline in the percentage of prescriptions filled by patients without known opioid tolerance persisted even when the TIRF-REMS–associated change in the rate of prescribing had diminished. Trends of prescribing to patients without known opioid tolerance using various lookback periods are shown in eFigure 13 in the Supplement. Sensitivity analyses found no brand-specific differences associated with TIRF-REMS implementation for Fentora, the only brand with enough prescriptions to analyze this outcome (eFigure 14 in the Supplement).

## Discussion

In this cohort study using interrupted time series analysis of TIRF prescribing for Medicare Part D beneficiaries between 2010 and 2014, we found that implementation of the TIRF-REMS Access Program, a mandatory restrictive drug distribution program, was associated with a temporary decline in the rate of overall TIRF prescribing, with a possible temporary decline in the percentage of TIRF prescribed to patients without cancer, as well as a lasting decrease in the percentage of TIRF prescribed to patients without known opioid tolerance. To our knowledge, this is the first study examining TIRF prescribing following TIRF-REMS implementation.

A closer review of the post-REMS implementation data reveals 2 distinct periods: the 12-month period following implementation, during which prescribing remained depressed relative to pre-REMS, followed by a period of prescribing increases that returned TIRF prescribing to near pre-REMS levels beginning mid-2013. One explanation for the observed attenuation of the decrease in prescribing associated with the program could be that it may have taken a year for patients, prescribers, and pharmacists to familiarize themselves with REMS requirements, and to register and complete the educational program. However, the subsequent return to baseline prescribing levels occurred despite a persistent decrease in the number of prescribers. This points to other possible contributing factors to the subsequent return of increased TIRF prescribing.

Notably, the increase in prescribing coincided with a marked increase in filled prescriptions for Subsys, a fentanyl sublingual spray that became the most commonly prescribed TIRF during this period. There have been reports that the Subsys manufacturer used aggressive,^5,19^ possibly illegal,^20,21^ strategies to increase drug sales and made efforts to guide prescribers and patients through the REMS program requirements.^6^ These efforts may have attenuated the prescribing decrease associated with TIRF-REMS implementation.

The overall decline in TIRF prescribing following TIRF-REMS implementation was associated with either similar or close to similar declines in prescription claims for both patients with and without cancer, depending on the cancer definition used. This highlights a potential unintended consequence of the TIRF-REMS.

Two additional study findings are notable. First, TIRF prescriptions represented only 0.03% of all opioid prescriptions, highlighting the relatively narrow scope of the TIRF-REMS program. Second, 79% of TIRF prescriptions were written for patients younger than 65 years, who typically receive Medicare benefits owing to disability or end-stage renal disease. These findings are consistent with previous studies that have shown a high rate of opioid prescribing in patients with end stage renal disease^22^ and Medicare disability beneficiaries^23^ and point to a population that may benefit from focused interventions.

### Limitations

Our study has important limitations. First, it is possible that unmeasured policy and/or clinical practice changes, such as the increasing use of prescription drug monitoring programs, or rescheduling of narcotic drug classes, impacted TIRF and other opioid prescribing. While time series methodology, along with the all-opioid control group we used, help mitigate external confounders, they do not eliminate them. Similarly, demographic shifts among Part D enrollees could have affected prescribing. While we did not have monthly demographic data for all Part D enrollees to include as covariates in the models, annual data show no notable year-to-year shifts in sex, race, age, or dual-eligible status of Part D enrollees.^24^ Second, we only had access to prescription data for Medicare beneficiaries who participate in Part D. Medicare Part D beneficiaries receive approximately 25% of all prescriptions written in the United States,^25^ but demographic differences may result in dissimilarities from national prescription rates. Furthermore, given the relatively small number of TIRF prescriptions for Part D beneficiaries, many of the TIRF brands did not have stable monthly prescription numbers to enable brand-level analysis for all outcomes measured. For those that did have enough prescriptions to enable analysis, we may not have had the statistical power to find significant brand-specific differences from generic. Third, our data only include prescriptions paid for by Medicare and do not include prescriptions paid for out of pocket, although the relatively high cost of TIRF drugs makes non–insurance coverage use less likely. Fourth, formulary changes could have affected prescribing. However, midyear, restrictive formulary changes are generally prohibited, so they are unlikely to have caused the immediate level changes noted after TIRF-REMS implementation, which occurred in March, although they may have affected the pretrend and posttrend changes. Fifth, the results from the 2-group time series analyses that compared all opioids to TIRFs may be biased because there were statistically significant differences in the preintervention level as well as trend.^14^ Still, even as an imperfect control, we believe all-opioid prescribing helps control for unmeasured confounders.

## Conclusions

We found that a mandatory educational and restrictive distribution program for TIRF products, the TIRF-REMS Access Program, was associated with a temporary decrease in overall TIRF prescribing, possibly with a temporary decrease in off-label TIRF prescribing, and with a lasting decline in the percentage of TIRF products prescribed to patients without known opioid tolerance.

## Notes

Supplement. **eAppendix 1.** Source and Processing of Prescribing Data **eAppendix 2.** Exclusions **eAppendix 3.** Interrupted Time Series Models **eAppendix 4.** Sensitivity Analysis Excluding Buprenorphine **eAppendix 5.** Model Adjustment **eAppendix 6.** Interpretation of Coefficients **eTable 1.** Medicare Part D Opioid Prescriptions, 2010-2014 **eTable 2.** Part D Transmucosal Immediate-Release Fentanyl Prescriptions by Brand, 2010-2014 **eTable 3.** Opioid Morphine Equivalent Conversion Factors Used for Study Morphine Milligram Equivalent (MME) Calculations **eFigure 1.** Adjusted Interrupted Time Series Models for TIRF Prescribing (Panel A) and All-Opioid Prescribing (Panel B), 2010-2014 **eFigure 2.** Adjusted Two-Group Interrupted Time Series Model of All-Opioid Prescriptions and Transmucosal Immediate-Release Fentanyl (TIRF) Prescriptions per 100,000 Part D Participants **eFigure 3.** Adjusted 2-Group Interrupted Time Series Model of Monthly Transmucosal Immediate-Release Fentanyl Prescribing, by Age Group **eFigure 4.** Adjusted, Interrupted Time Series Models of Transmucosal Immediate-Release Fentanyl Prescribing, 2010-2014, by Brand **eFigure 5.** Post-hoc Analysis of the Three Primary Outcomes With and Without Subsys-Brand Transmucosal Immediate-Release Fentanyl (TIRF) Prescriptions **eFigure 6.** Monthly Transmucosal Immediate-Release Fentanyl Prescriptions and Prescribers per 100,000 Part D Beneficiaries, 2010-2014 **eFigure 7.** Adjusted Time Series Models of Monthly Transmucosal Immediate-Release Fentanyl (TIRF) Prescribing to Patients Without Cancer (Panel A) and With Cancer (Panel B), 2010-2014 **eFigure 8.** Two-Group Adjusted Interrupted Time Series Model of Monthly Transmucosal Immediate-Release Fentanyl Scripts per 100,000 Part D Beneficiaries by Cancer Status **eFigure 9.** Adjusted Interrupted Time Series Models of the Monthly Percentage of Transmucosal Immediate-Release Fentanyl Prescriptions Filled by Patients Without Cancer **eFigure 10.** Adjusted Interrupted Time Series Models of Transmucosal Immediate-Release Fentanyl Prescribing to Patients Without Cancer, 2010-2014, by Brand **eFigure 11.** Monthly Percentage of Transmucosal Immediate-Release Fentanyl (TIRF) Prescriptions Filled by Patients Without Cancer Diagnosis, by TIRF Brand **eFigure 12.** Adjusted Interrupted Time Series Model of Monthly Percentage of Transmucosal Immediate-Release Fentanyl Prescriptions to Patients Without Known Opioid Tolerance, 2010-2014 **eFigure 13.** Percent of Transmucosal Immediate-Release Fentanyl Prescriptions to Patients Without Known Opioid Tolerance Using Various Lookback Periods, 2010-2014 **eFigure 14.** Adjusted Interrupted Time Series Model of the Monthly Percentage of Generic vs Fentora-Branded Transmucosal Immediate-Release Fentanyl Prescriptions to Patients Without Opioid Tolerance, 2010-2014 **eReferences**
