# Supplementary material for: Association of a Risk Evaluation and Mitigation Strategy Program With Transmucosal Fentanyl Prescribing
Source: JAMA Netw Open. 2019 Mar 29;2(3):e191340. doi: 10.1001/jamanetworkopen.2019.1340 (PMC6450314; doi:10.1001/jamanetworkopen.2019.1340)
Supplement: Supplement. — eAppendix 1. Source and Processing of Prescribing Data eAppendix 2. Exclusions eAppendix 3. Interrupted Time Series Models eAppendix 4. Sensitivity Analysis Excluding Buprenorphine eAppendix 5. Model Adjustment eAppendix 6. Interpretation of Coefficients eTable 1. Medicare Part D Opioid Prescriptions, 2010-2014 eTable 2. Part D Transmucosal Immediate-Release Fentanyl Prescriptions by Brand, 2010-2014 eTable 3. Opioid Morphine Equivalent Conversion Factors Used for Study Morphine Milligram Equivalent (MME) Calculations eFigure 1. Adjusted Interrupted Time Series Models for TIRF Prescribing (Panel A) and All-Opioid Prescribing (Panel B), 2010-2014 eFigure 2. Adjusted Two-Group Interrupted Time Series Model of All-Opioid Prescriptions and Transmucosal Immediate-Release Fentanyl (TIRF) Prescriptions per 100,000 Part D Participants eFigure 3. Adjusted 2-Group Interrupted Time Series Model of Monthly Transmucosal Immediate-Release Fentanyl Prescribing, by Age Group eFigure 4. Adjusted, Interrupted Time Series Models of Transmucosal Immediate-Release Fentanyl Prescribing, 2010-2014, by Brand eFigure 5. Post-hoc Analysis of the Three Primary Outcomes With and Without Subsys-Brand Transmucosal Immediate-Release Fentanyl (TIRF) Prescriptions eFigure 6. Monthly Transmucosal Immediate-Release Fentanyl Prescriptions and Prescribers per 100,000 Part D Beneficiaries, 2010-2014 eFigure 7. Adjusted Time Series Models of Monthly Transmucosal Immediate-Release Fentanyl (TIRF) Prescribing to Patients Without Cancer (Panel A) and With Cancer (Panel B), 2010-2014 eFigure 8. Two-Group Adjusted Interrupted Time Series Model of Monthly Transmucosal Immediate-Release Fentanyl Scripts per 100,000 Part D Beneficiaries by Cancer Status eFigure 9. Adjusted Interrupted Time Series Models of the Monthly Percentage of Transmucosal Immediate-Release Fentanyl Prescriptions Filled by Patients Without Cancer eFigure 10. Adjusted Interrupted Time Series Models of Transmucosal Immediate-Release Fentanyl [file jamanetwopen-2-e191340-s001.pdf]

# Supplementary Online Content

Fleischman W, Auth D, Shah ND, Agrawal S, Ross JS. Association of a risk evaluation and mitigation strategy program with transmucosal fentanyl prescribing. *JAMA Netw Open*. 2019;2(3):e191340. doi:10.1001/jamanetworkopen.2019.1340

**eAppendix 1.** Source and Processing of Prescribing Data

**eAppendix 2.** Exclusions

**eAppendix 3.** Interrupted Time Series Models

**eAppendix 4.** Sensitivity Analysis Excluding Buprenorphine

**eAppendix 5.** Model Adjustment

**eAppendix 6.** Interpretation of Coefficients

**eTable 1.** Medicare Part D Opioid Prescriptions, 2010-2014

**eTable 2.** Part D Transmucosal Immediate-Release Fentanyl Prescriptions by Brand, 2010-2014

**eTable 3.** Opioid Morphine Equivalent Conversion Factors Used for Study Morphine Milligram Equivalent (MME) Calculations

**eFigure 1.** Adjusted Interrupted Time Series Models for TIRF Prescribing (Panel A) and All-Opioid Prescribing (Panel B), 2010-2014

**eFigure 2.** Adjusted Two-Group Interrupted Time Series Model of All-Opioid Prescriptions and Transmucosal Immediate-Release Fentanyl (TIRF) Prescriptions per 100,000 Part D Participants

**eFigure 3.** Adjusted 2-Group Interrupted Time Series Model of Monthly Transmucosal Immediate-Release Fentanyl Prescribing, by Age Group

**eFigure 4.** Adjusted, Interrupted Time Series Models of Transmucosal Immediate-Release Fentanyl Prescribing, 2010-2014, by Brand

**eFigure 5.** Post-hoc Analysis of the Three Primary Outcomes With and Without Subsys-Brand Transmucosal Immediate-Release Fentanyl (TIRF) Prescriptions

**eFigure 6.** Monthly Transmucosal Immediate-Release Fentanyl Prescriptions and Prescribers per 100,000 Part D Beneficiaries, 2010-2014

**eFigure 7.** Adjusted Time Series Models of Monthly Transmucosal Immediate-Release Fentanyl (TIRF) Prescribing to Patients Without Cancer (Panel A) and With Cancer (Panel B), 2010-2014

© 2019 Fleischman W et al. *JAMA Network Open.*

**eFigure 8.** Two-Group Adjusted Interrupted Time Series Model of Monthly Transmucosal Immediate-Release Fentanyl Scripts per 100,000 Part D Beneficiaries by Cancer Status

**eFigure 9.** Adjusted Interrupted Time Series Models of the Monthly Percentage of Transmucosal Immediate-Release Fentanyl Prescriptions Filled by Patients Without Cancer

**eFigure 10.** Adjusted Interrupted Time Series Models of Transmucosal Immediate-Release Fentanyl Prescribing to Patients Without Cancer, 2010-2014, by Brand

**eFigure 11.** Monthly Percentage of Transmucosal Immediate-Release Fentanyl (TIRF) Prescriptions Filled by Patients Without Cancer Diagnosis, by TIRF Brand

**eFigure 12.** Adjusted Interrupted Time Series Model of Monthly Percentage of Transmucosal Immediate-Release Fentanyl Prescriptions to Patients Without Known Opioid Tolerance, 2010-2014

**eFigure 13.** Percent of Transmucosal Immediate-Release Fentanyl Prescriptions to Patients Without Known Opioid Tolerance Using Various Lookback Periods, 2010-2014

**eFigure 14.** Adjusted Interrupted Time Series Model of the Monthly Percentage of Generic vs Fentora-Branded Transmucosal Immediate-Release Fentanyl Prescriptions to Patients Without Opioid Tolerance, 2010-2014

**eReferences**

This supplementary material has been provided by the authors to give readers additional information about their work.

***eAppendix 1. Source and processing of prescribing data***

We obtained line-item prescription claims data for all drugs classified as opioids from the Chronic Condition Warehouse using the group "65" identifier in the Master Drug Database classification system, which is one of the classification systems the Warehouse uses to categorize drug claims. The data included anonymized beneficiary identifiers, date of prescription dispensation, brand and generic drug names, dose, quantity dispensed, and prescriber NPI. We linked the pharmacy claims to limited beneficiary-level demographic and health data using these anonymized beneficiary identifiers.

***eAppendix 2. Exclusions***

For all analyses, we excluded 229,705 prescription claims (0.06% of all prescriptions) for injectable opioids, and 98 prescriptions claims that were missing the route.

For the time series analyses of overall TIRF prescribing and prescribing to patients without cancer, we also excluded prescriptions for sublingual fentanyl tablets marketed as Abstral, fentanyl nasal spray marketed as Lazanda, and fentanyl buccal film marketed as Onsolis (0.96%, 0.49%, and 0.24% of all TIRF prescriptions, respectively), as these drugs had separate REMS implemented prior to the class-wide TIRF implementation. We did, however, include Abstral and Onsolis prescriptions in the analyses of prescribing to patients without known opioid tolerance since these prescriptions could have affected patients' tolerance status, and also included them in the descriptive analyses to provide a complete overview of Part D TIRF prescribing.

For the by-brand analysis for each of the primary outcomes, the only brands that had prescriptions during each month of the study period to allow for brand-level analysis were Actiq and Fentora, and only Fentora had enough prescriptions to enable brand-level analysis on prescribing to patients without known opioid tolerance. Additionally, for the time series analysis of percentage of prescribing to patients without known opioid tolerance, we excluded the first 3 months of 2010, since we used a 90 day look-back period to establish patients' prescribing history and opioid tolerance.

© 2019 Fleischman W et al. *JAMA Network Open.*

***eAppendix 3. Interrupted Time Series Models***

We performed interrupted time series analyses using segmented ordinary least squares regression[1] with robust Newey-West errors to account for autocorrelation and heteroskedasticity.[2] This method of analysis calculates independent tests of the *level* (the intercept) and *trend* (slope) before and after an interruption, or intervention, and then evaluates for differences between the levels and slopes.

For single group analyses, the model is represented by the following figure and equation:

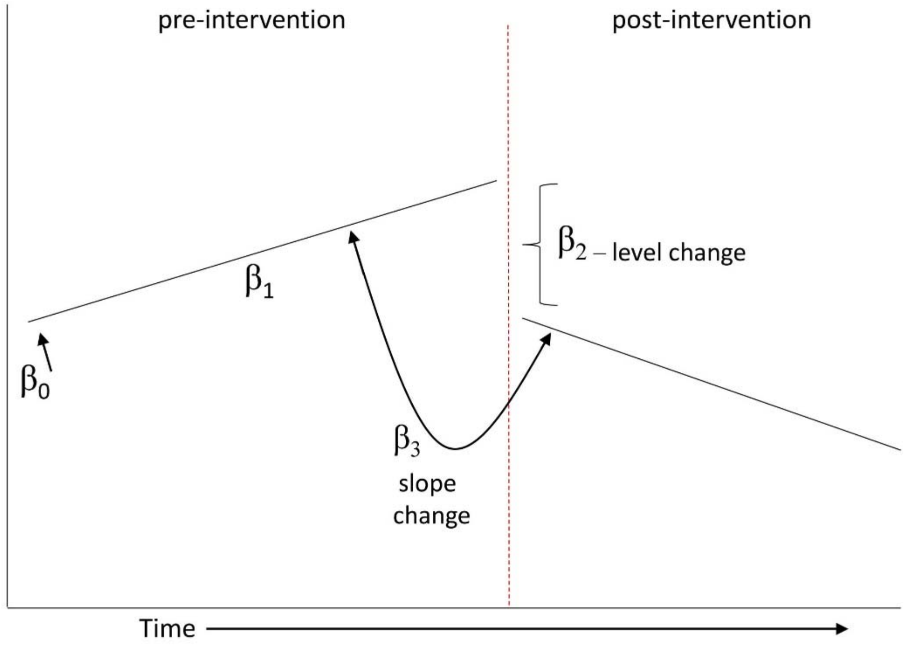


$$Yt = \beta_0 + \beta_1 T + \beta_2 X_t + \beta_3 X_{tt} + \varepsilon_t$$

$Y_t$ is the aggregated outcome variable measured at each time-point t; $T_t$ is the time since the start of the study; $X_t$ is a dummy variable representing the intervention; $X_tT_t$ is an interaction term; $\beta_0$ represents the intercept, or starting level of the outcome variable; $\beta_1$ is the slope of the outcome variable until the introduction of the intervention; $\beta_2$ represents the change in the level of the outcome that occurs in the period immediately following the introduction of the intervention; $\beta_3$ represents the difference between pre and post-intervention slopes of the outcome.[2]

© 2019 Fleischman W et al. *JAMA Network Open.*

For multiple group analyses, the figure and equation are as follows:

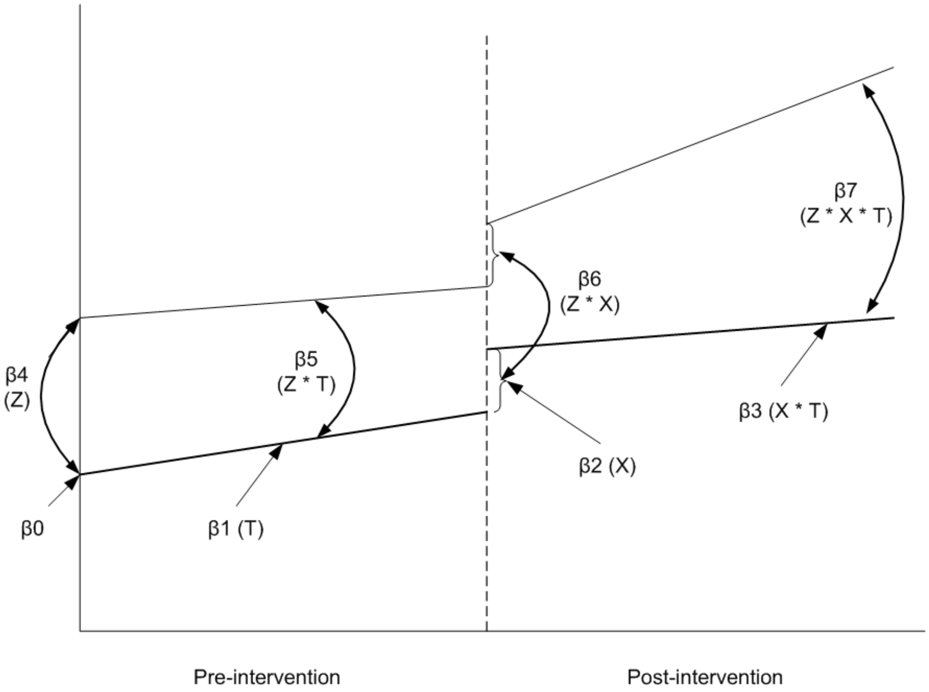


*Figure originally published in the Stata Journal (volume 15: 482)[2] and is used with the permission of the author and StataCorp.

$$Yt = \beta_0 + \beta_1 T_t + \beta_2 X_t + \beta_3 X_t T_t + \beta_4 Z + \beta_5 Z T_t + \beta_6 Z X_t + \beta_7 Z X_t T_t + \varepsilon_t$$

In this model, Z is a dummy variable denoting the treatment/control groups; $ZT_t$, $ZX_t$ and $ZX_tT_t$ are all interaction terms. In the figure below: $\beta_0$ to $\beta_3$ represent the control group; $\beta_4$ to $\beta_7$ represent the treatment group. Specifically: $\beta_4$ represents the difference in the *level* or intercept of the outcome variable between treatment and controls prior to the intervention, while $\beta_5$ represents the difference in the *slope* or trend of the outcome variable between treatment and controls prior to the intervention. $\beta_6$ indicates the difference between treatment and control groups in the *level* of the outcome variable immediately following introduction of the intervention, and $\beta_7$ represents the difference between treatment and control groups in the *slope* of the outcome variable after initiation of the intervention compared to pre-intervention (akin to a difference-in-differences of slopes).[2,4]

***eAppendix 4. Sensitivity analysis excluding buprenorphine***

As noted in the manuscript, we performed sensitivity analyses to evaluate for potential confounding by factors that may have affected all opioid prescribing, such as increasing national attention to prescription opioid harms. To do this, we performed multiple group time series analyses, using all-opioid prescriptions as a control group. We first included all opioids, including opioid drugs commonly used for medication assisted therapy (MAT) for opioid use disorders such as methadone and buprenorphine, as well as medications generally marketed for cough and cold treatment that contain opioids. Notably, Medicare Part D does not cover methadone for MAT, but does provide coverage for methadone when prescribed for pain, and there were more than 7 million filled prescriptions for methadone during our study period (**eTable 2**). Part D does cover buprenorphine-containing medications. Since it is possible that an increase in buprenorphine prescribing for MAT could have masked a decrease in all-opioid prescribing (thus confounding our use of all opioid prescriptions as a control), we repeated the above analyses while excluding buprenorphine-containing drugs. Results are as noted in **eFigure 2a**.

***eAppendix 5. Model adjustment***

We tested for autocorrelation up to 6 "lags", or time periods, in the error distribution using the Cumby-Huizinga test, and specified the models to ensure correct autocorrelation structures. Initial examination of the data showed seasonal variation, with increases in prescribing around the beginning of the year. We therefore included a variable denoting the months of December and January. We also included a variable denoting the number of days in each month of the study.

***eAppendix 6. Interpretation of coefficients***

The study results included both absolute number changes (e.g. monthly TIRF prescriptions per 100,000 Part D participants), as well as percentage point changes (e.g. monthly percentage of TIRF prescriptions for patients without cancer). For ease of interpretation, we calculated and report relative percent changes for all outcomes. We calculated relative percent changes by log transforming the dependent variable for all analyses, and then exponentiating the resulting coefficients.

© 2019 Fleischman W et al. *JAMA Network Open.*

**eTable 1**. Medicare Part D Opioid prescriptions, 2010-2014.

| Drug, by active ingredient(s) | No. prescriptions |
|---|---|
| acetaminophen with codeine | 13,574,255 |
| Buprenorphine | 564,358 |
| buprenorphine HCL/naloxone HCL | 2,141,250 |
| butorphanol tartrate | 230,458 |
| codeine sulfate | 147,243 |
| codeine/butalbital/acetaminophen/caffeine | 385,094 |
| codeine/butalbital/aspirin/caffeine | 385,343 |
| dihydrocodeine/acetaminophen/caffeine | 23,492 |
| dihydrocodeine/aspirin/caffeine | 459 |
| Fentanyl | 14,798,544 |
| fentanyl citrate | 82,086 |
| hydrocodone/acetaminophen | 161,267,554 |
| hydrocodone bitartrate | 5,270 |
| hydrocodone/ibuprofen | 1,316,635 |
| hydromorphone HCL | 5,165,865 |
| ibuprofen/oxycodone HCL | 3,928 |
| levorphanol tartrate | 8,579 |
| meperidine HCL | 309,639 |
| methadone HCL | 7,080,584 |
| morphine sulfate | 18,375,047 |
| morphine sulfate/naltrexone | 15,886 |
| oxycodone HCL | 32,258,378 |
| oxycodone HCL/acetaminophen | 44,321,770 |
| oxycodone HCL/aspirin | 63,331 |
| oxycodone HCL/oxycodone terephthalate/aspirin | 38,156 |
| oxymorphone HCL | 1,724,331 |
| pentazocine HCL/acetaminophen | 35,166 |
| pentazocine HCL/naloxone HCL | 143,162 |
| propoxyphene HCL | 119,249 |
| propoxyphene napsylate | 6,596 |
| propoxyphene/acetaminophen | 4,486,133 |
| tapentadol HCL | 468,704 |
| tramadol HCL | 57,088,722 |
| tramadol HCL/acetaminophen | 5,388,052 |
| Total | **372,023,319** |

© 2019 Fleischman W et al. *JAMA Network Open.*

**eTable 2**. Part D transmucosal immediate-release fentanyl prescriptions by brand, 2010-2014.

| Brand | No. prescriptions |
|---|---|
| Generic fentanyl citrate | 56,259 |
| Fentora | 20,036 |
| Subsys | 17,515 |
| Actiq | 4,111 |
| Abstral | 957 |
| Lazanda | 486 |
| Onsolis | 237 |
| Total | **99,601** |

© 2019 Fleischman W et al. *JAMA Network Open.*

**eTable 3.** Opioid morphine equivalent conversion factors used for study morphine milligram equivalent (MME) calculations.

| Type of Opioid | MME Conversion Factor |
|---|---|
| Buprenorphine patch | 12.6 |
| Buprenorphine tab or film | 10 |
| Butorphanol | 7 |
| Codeine | 0.15 |
| Dihydrocodeine | 0.25 |
| Fentanyl buccal or SL tablets, or lozenge/troche | 0.13 |
| Fentanyl film or oral spray | 0.18 |
| Fentanyl nasal spray | 0.16 |
| Fentanyl patch | 7.2 |
| Hydrocodone | 1 |
| Hydromorphone | 4 |
| Levorphanol tartrate | 11 |
| Meperidine hydrochloride | 0.1 |
| Methadone | 3 |
| Morphine | 1 |
| Nalbuphine | 1 |
| Opium | 1 |
| Oxycodone | 1.5 |
| Oxymorphone | 3 |
| Pentazocine | 0.37 |
| Tapentadol | 0.4 |
| Tramadol | 0.1 |

Source: https://www.cms.gov/Medicare/Prescription-Drug-Coverage/PrescriptionDrugCovContra/Downloads/Opioid-Morphine-EQ-Conversion-Factors-March-2015.pdf

© 2019 Fleischman W et al. *JAMA Network Open.*

**eFigure 1.** Adjusted interrupted time series models for TIRF prescribing **(Panel A)** and all-opioid prescribing **(Panel B)**, 2010-2014. Points represent the raw data and lines represent the adjusted, best fit slope. Dotted line denotes TIRF-REMS implementation in March 2012.

**A**

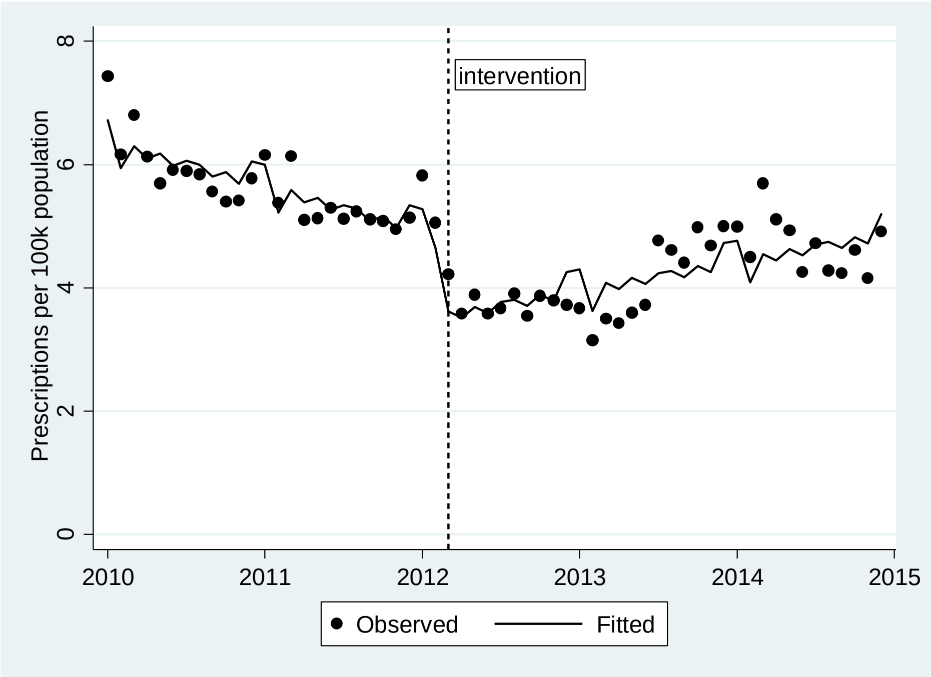


**B**

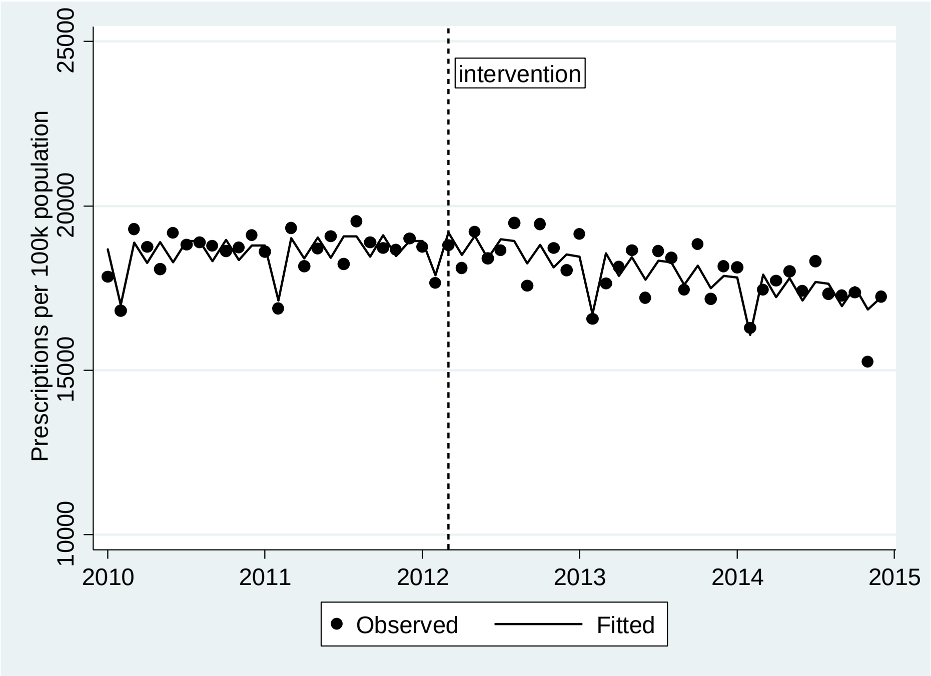

**eFigure 2.** Adjusted two-group interrupted time series model of all-opioid prescriptions and transmucosal immediate-release fentanyl (TIRF) prescriptions per 100,000 Part D participants. We use the log of prescriptions to compare the two on a similar scale since opioid prescriptions vastly outnumber TIRF prescriptions. Compared to all-opioid prescribing, TIRF prescribing had a monthly decrease of 1.1% during the pre-intervention period (95% CI, -1.6, -0.57, p<0.001), a level decrease upon REMS implementation of 27.6% (95% CI, -34.1, -20.6, p<0.001), and a post-REMS monthly increase in prescribing of 2.36% (95% CI, 1.63, 3.09, p<0.001). A sensitivity analysis that excluded buprenorphine prescriptions from the all-opioid control group showed similar results. Dashed vertical line denotes TIRF-REMS implementation in March 2012. Points represent the raw data and lines represent the adjusted, best fit slopes.

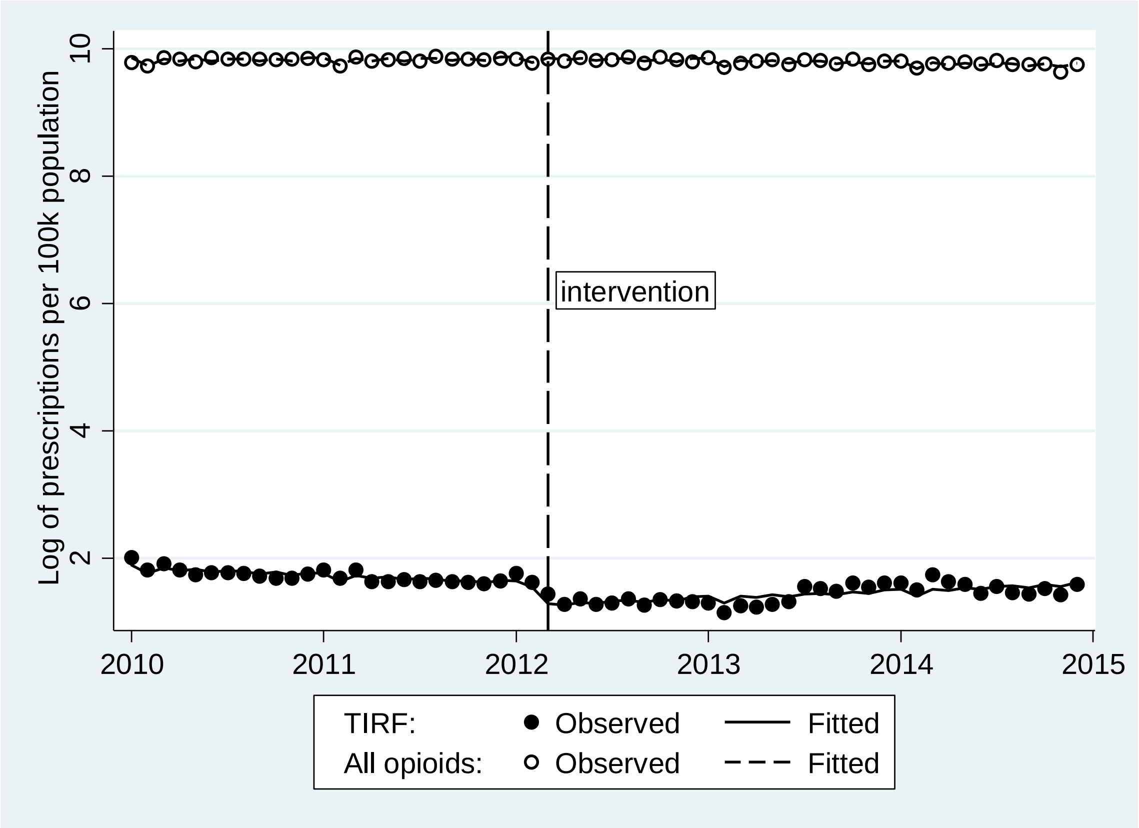


Abbrev: TIRF: and transmucosal immediate-release fentanyl

**eFigure 3**. Adjusted 2-group interrupted time series model of monthly transmucosal immediate-release fentanyl prescribing, by age group: patients younger than 65 (solid dots), and those 65 and older (circles). The outcome variable is the log of monthly TIRF prescriptions per 100,000 Part D participants. Compared to TIRF prescriptions for patients older than 65, there were 1% monthly decreases in TIRF prescriptions for patients younger than 65 during the pre-intervention period (95% CI, 1.8, 0.26; p=0.009), but there was no significant difference between the age groups in the level change (0.43%, 95% CI, -13.4, 14.5; p=0.95) or trend change (0.35%, 95% CI -1.42, 0.72; p=0.52) after TIRF-REMS implementation. Dotted vertical line denotes TIRF-REMS implementation in March 2012. Points represent the raw data and lines represent the adjusted, best fit slopes.

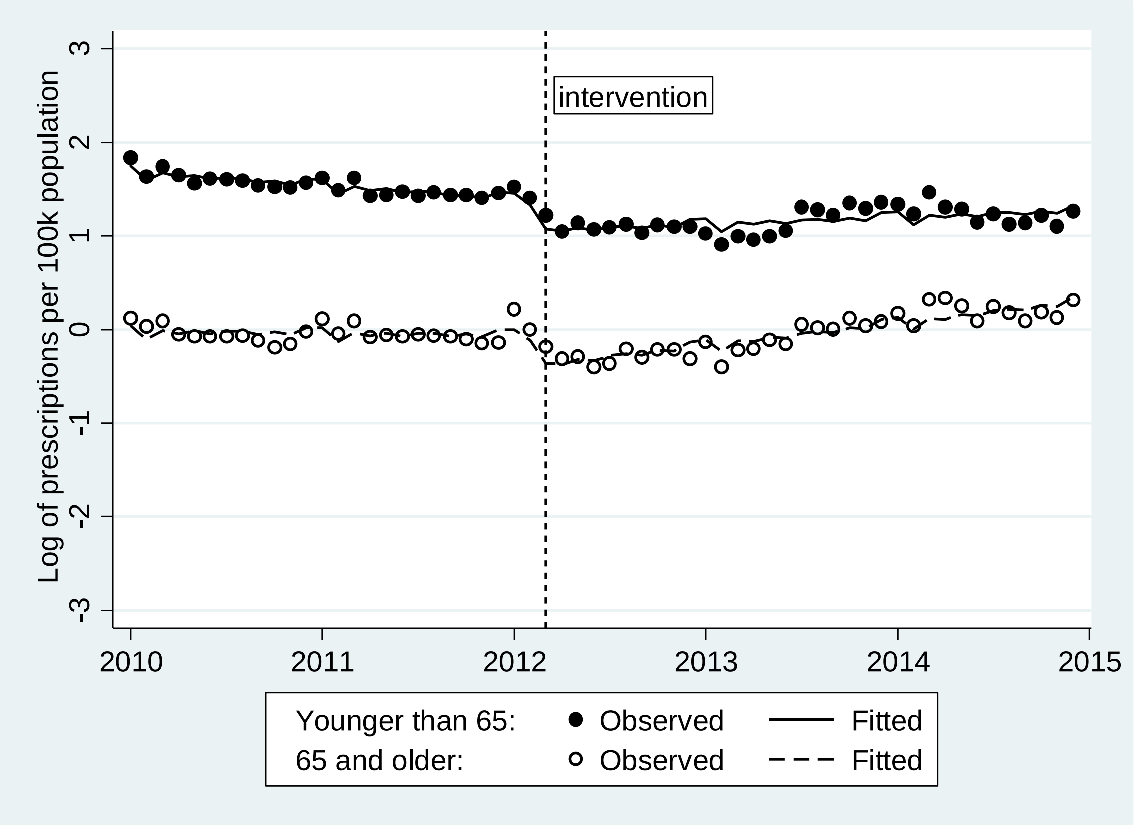

**eFigure 4**. Adjusted, interrupted time series models of transmucosal immediate-release fentanyl prescribing, 2010-2014, by brand: Actiq vs generic (**Panel A**) and Fentora vs generic (**Panel B**). When compared to generic prescriptions, there were no significant differences in the pre-TIRF-REMS trend among Actiq prescriptions (0.49%, 95% CI, -1.14, 0.16; p=0.14), nor in the level change (7.92%, 95% CI, -19.9, 5.81; p=0.24) or trend change (0.38%, 95% CI, -1.2, 0.46; p=0.38) after TIRF-REMS implementation. For Fentora, the pre-TIRF-REMS trend did differ from the trend for generics (1.2%, 95% CI, 0.6, 1.8; p<0.001), but there were no significant differences from generics in the level change (3.4%, 95% CI, -9.18, 17.8; p=0.61) or trend change (0.31%, 95% CI, -0.49, 1.1; p=0.44) after TIRF-REMS implementation. Dotted vertical lines denote TIRF-REMS implementation in March 2012. Points represent the raw data and lines represent the adjusted, best fit slopes.

**A**

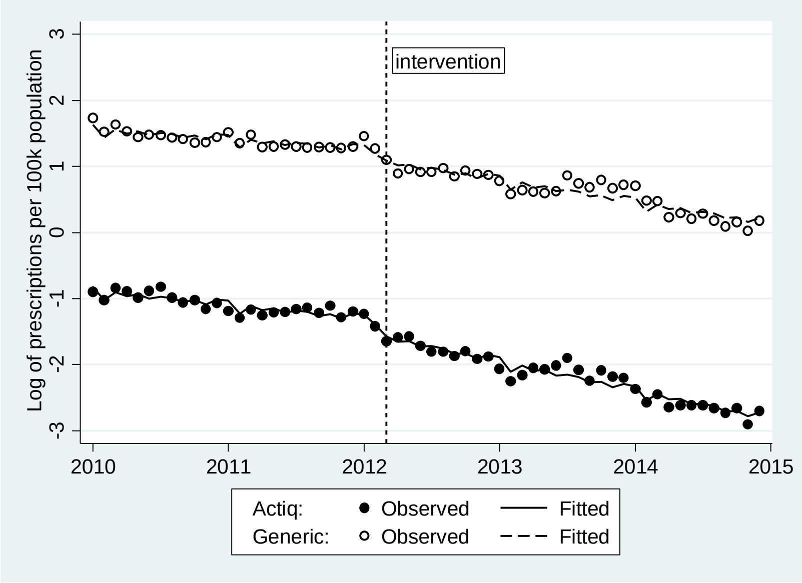


**B**

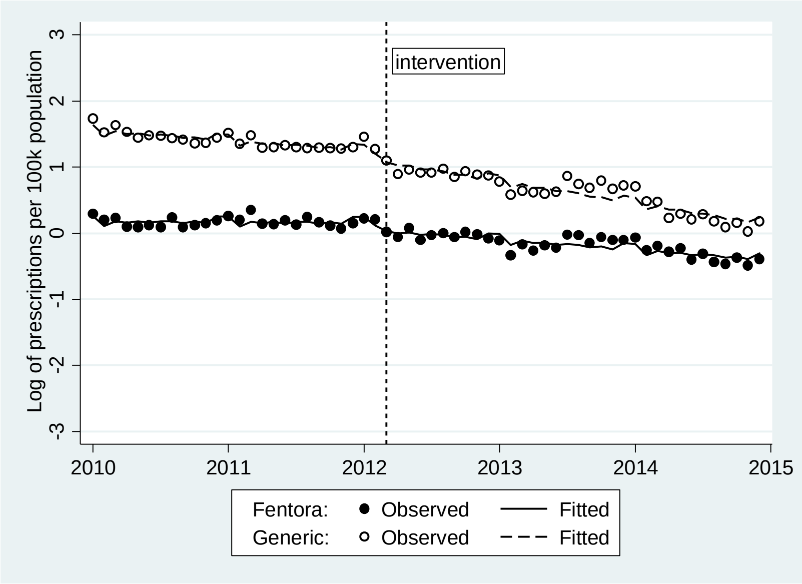

**eFigure 5.** Post-hoc analysis of the three primary outcomes with and without Subsys-brand transmucosal immediate-release fentanyl (TIRF) prescriptions: overall rate of TIRF prescribing, the percentage of TIRF prescriptions for patients without cancer, and the percentage of TIRF prescriptions for patients without known tolerance. This analysis is intended for hypothesis generation only. Interpretation is limited by the fact that there is no way to separate prescriptions that were 'converted' from generic to Subsys (following the introduction of Subsys to the market) vs those that were newly 'induced' by the manufacturer of Subsys through marketing and promotion.

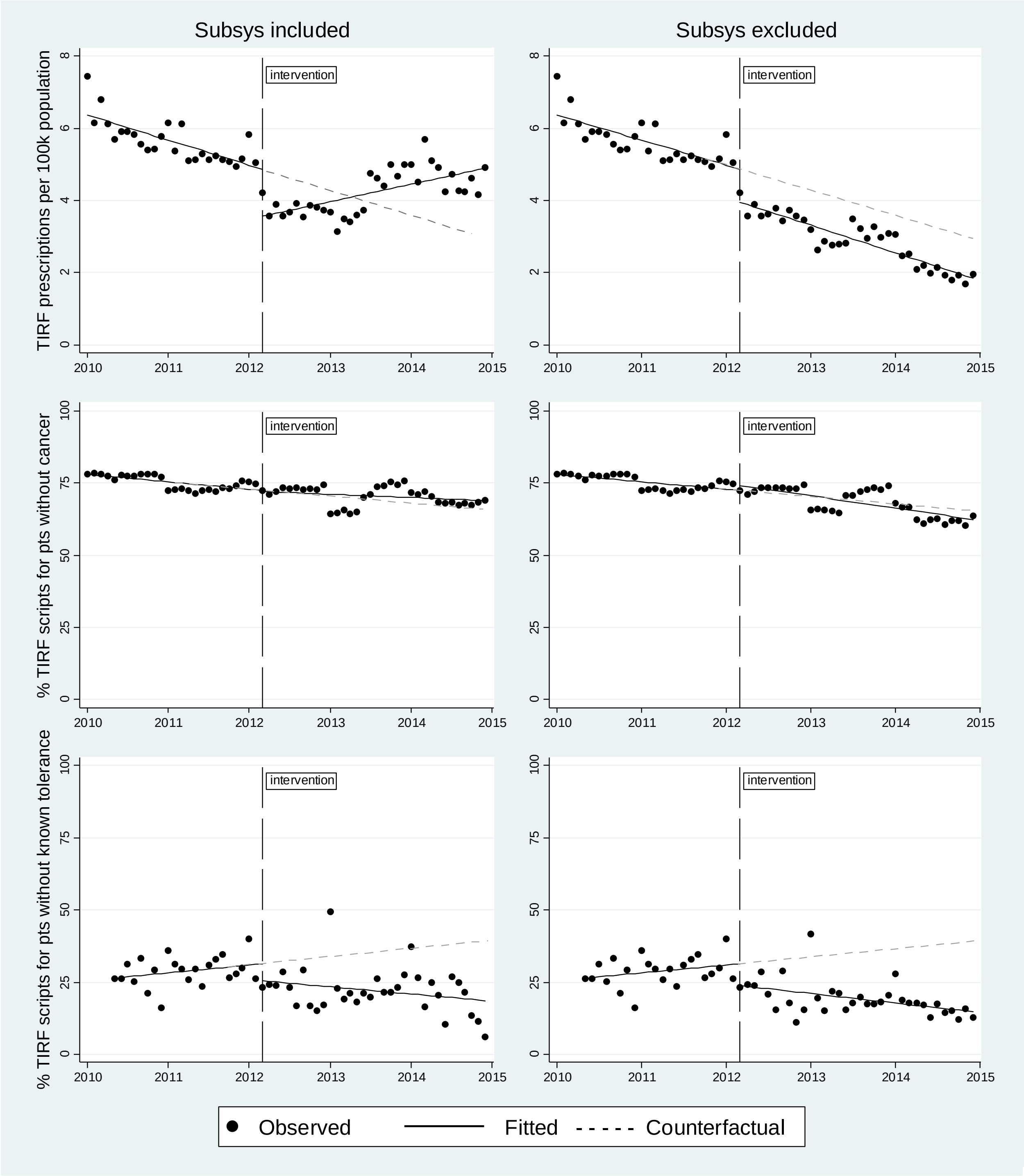

**eFigure 6.** Monthly transmucosal immediate-release fentanyl prescriptions and prescribers per 100,000 Part D beneficiaries, 2010-2014. Dotted line denotes month of TIRF-REMS implementation.

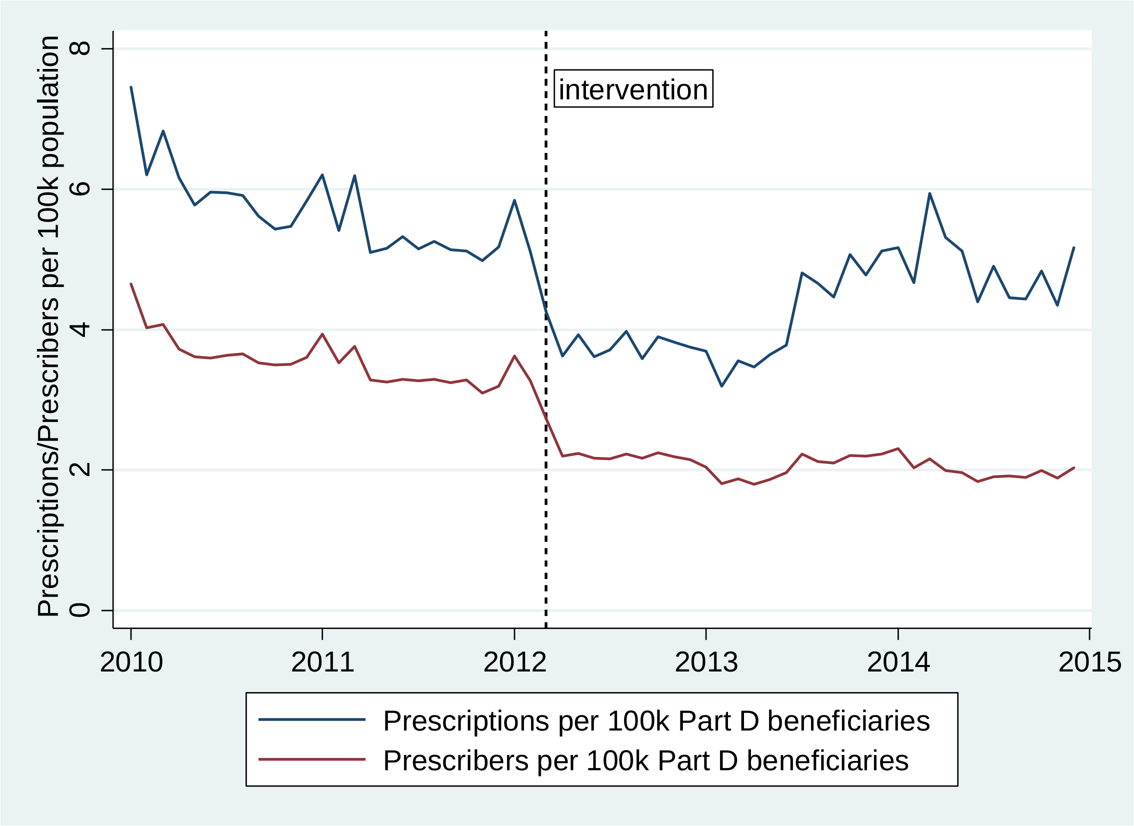

**eFigure 7.** Adjusted time series models of monthly transmucosal immediate-release fentanyl (TIRF) prescribing to pts without cancer (**Panel A**) and with cancer (**Panel B**), 2010-2014. This analysis uses the study's narrow cancer definition (claim with cancer diagnosis during prescription year). Declines in the level of TIRF prescribing after TIRF-REMS implementation were similar for patients without cancer (-27%, 95 CI%, -36.1.0, -16.6; p<0.001) to those with cancer diagnoses during the calendar year of the prescription (-27%, 95CI%, -34.0, -19.0; p<0.001). Dotted line denotes TIRF-REMS implementation in March 2012. Points represent the raw data and lines represent the adjusted, best fit slopes.

**A**

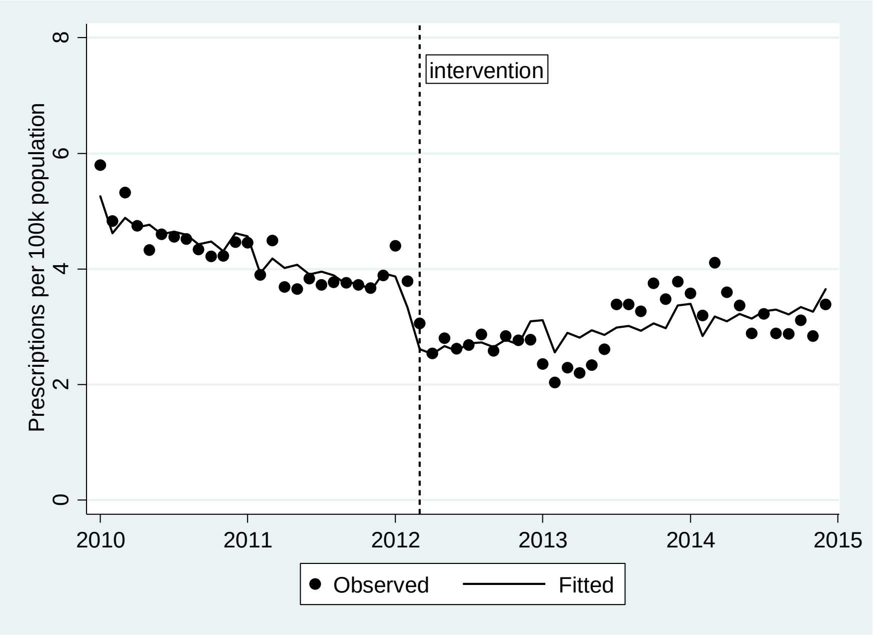


**B**

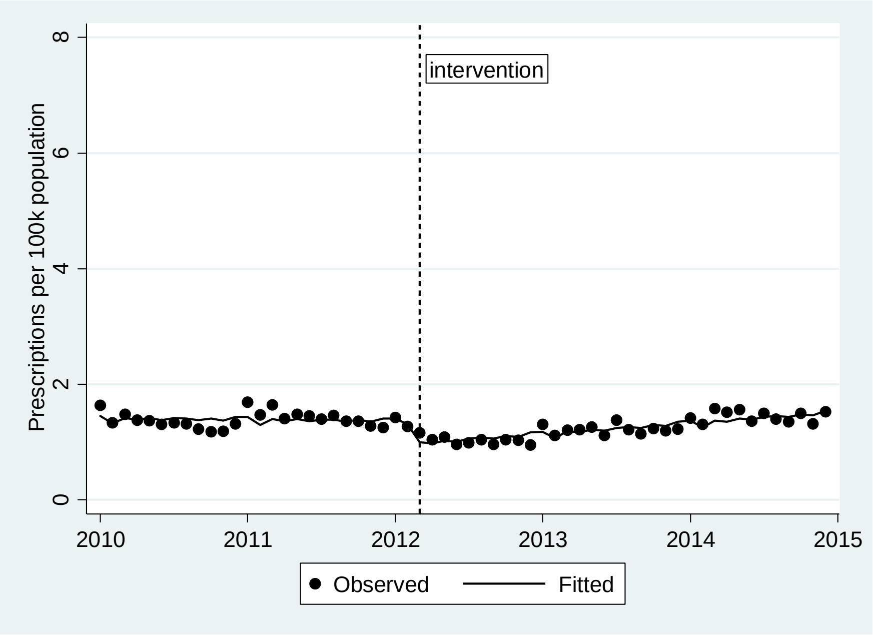

**eFigure 8.** Two-group adjusted interrupted time series model of monthly transmucosal immediate-release fentanyl scripts per 100,000 Part D beneficiaries by cancer status. No differences were found in the level change (0.87%, 95% CI -15.8, 16.7; p=0.92) or trend (0.72%, 95% CI, -0.27, 1.71; p=0.15) between the groups following TIRF-REMS implementation. Dashed vertical line denotes TIRF-REMS implementation in March 2012. Points represent the raw data and lines represent the adjusted, best fit slopes.

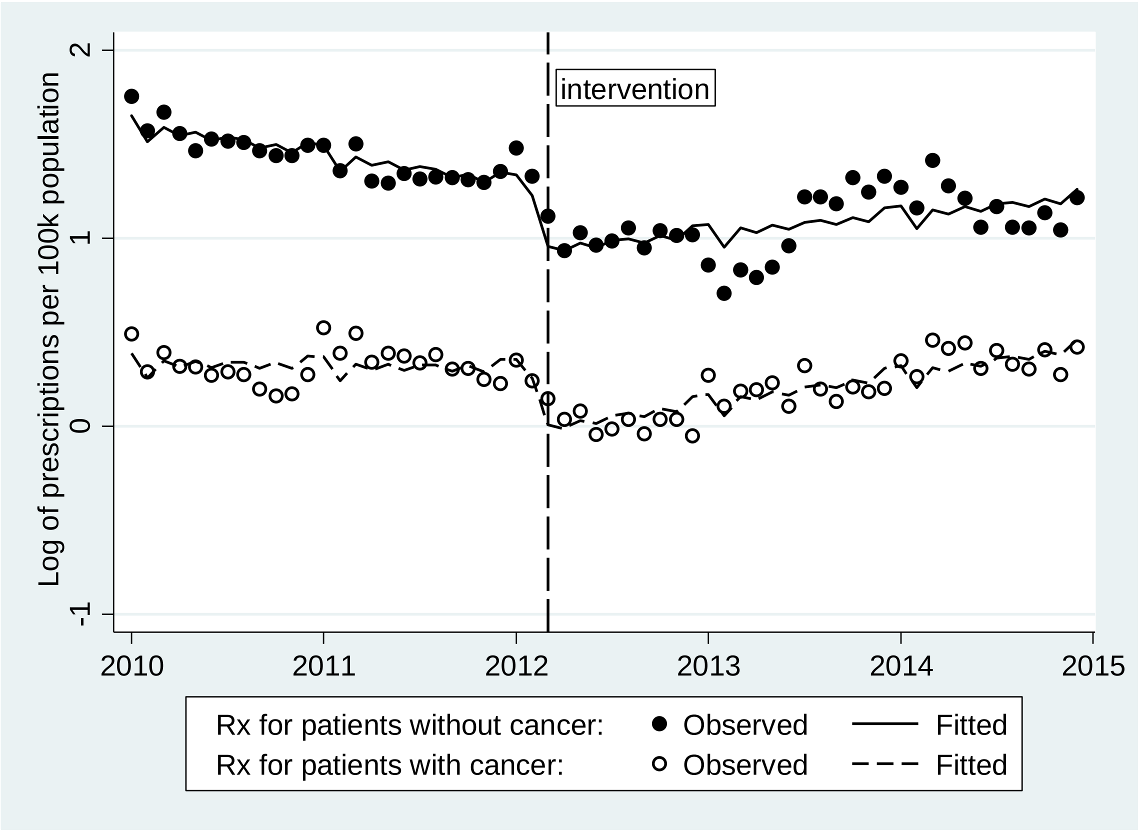

**eFigure 9.** Adjusted interrupted time series models of the monthly percentage of transmucosal immediate-release fentanyl prescriptions filled by patients without cancer. Cancer is defined either as the patient having a claim with a cancer diagnosis in the same calendar year as the filled prescription (**Panel A**), or as having a claim with a cancer diagnosis during any year of the study period (**Panel B)**. Repeat analysis using imputed values for outlier months in early 2013 showed similar results. Dotted line denotes TIRF-REMS implementation in March 2012. Points represent the raw data and lines represent the adjusted, best fit slopes.

**A**

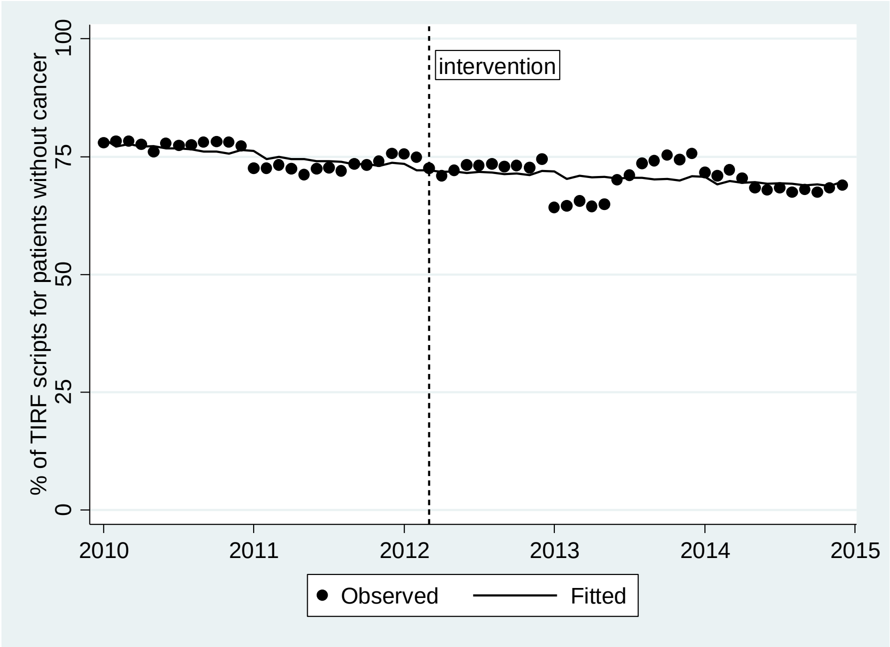


**B**

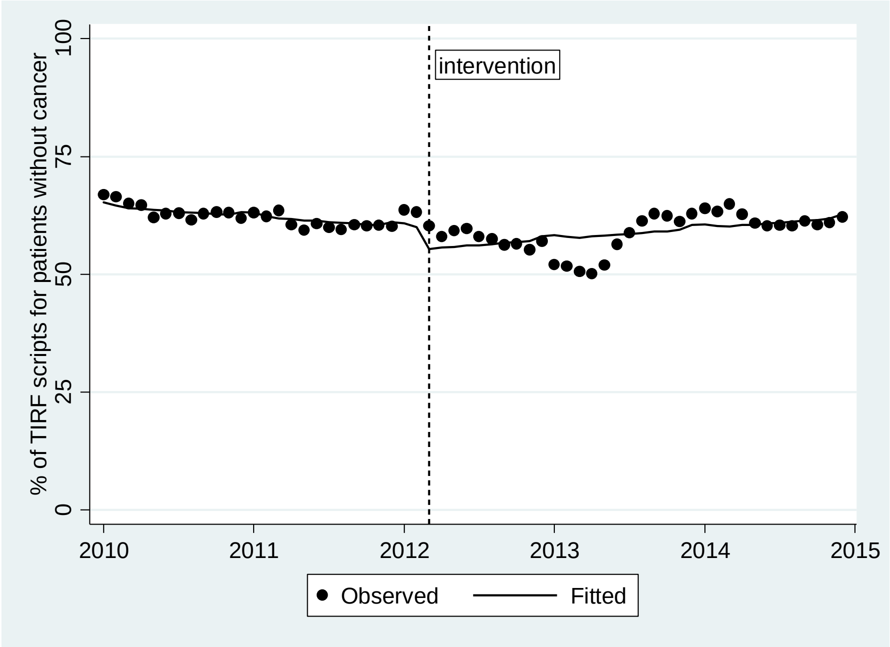

**eFigure 10**. Adjusted interrupted time series models of transmucosal immediate-release fentanyl prescribing to patients without cancer, 2010-2014, by brand: Actiq vs generic (**Panel A**), and Fentora vs generic (**Panel B**). Compared to generic prescriptions, there were no significant differences in the percentage of TIRF prescribed for patients without cancer in the pre-TIRF-REMS trend among Actiq prescriptions (0.25%, 95% CI, -0.004, 0.51; p=0.054), nor in the level (7.1%, 95% CI 14.4, 0.86; p=0.08), or trend (0.16%, 95% CI, -0.30, 0.61; p=0.49) after TIRF-REMS implementation. For Fentora, there was no significant difference in the pre-TIRF-REMS trend (0.03%, 95% CI, -0.24, 0.31, p=0.82); post implementation, there was an 8.3% level decline in prescriptions compared to generic (95% CI, 14.8, 1.27, p=0.02), with no difference in post-implementation trend (2.4%, 95% CI, -1.10, 0.59; p=0.18). Dotted vertical line denotes TIRF-REMS implementation in March 2012. Points represent the raw data and lines represent the adjusted, best fit slopes.

**A**

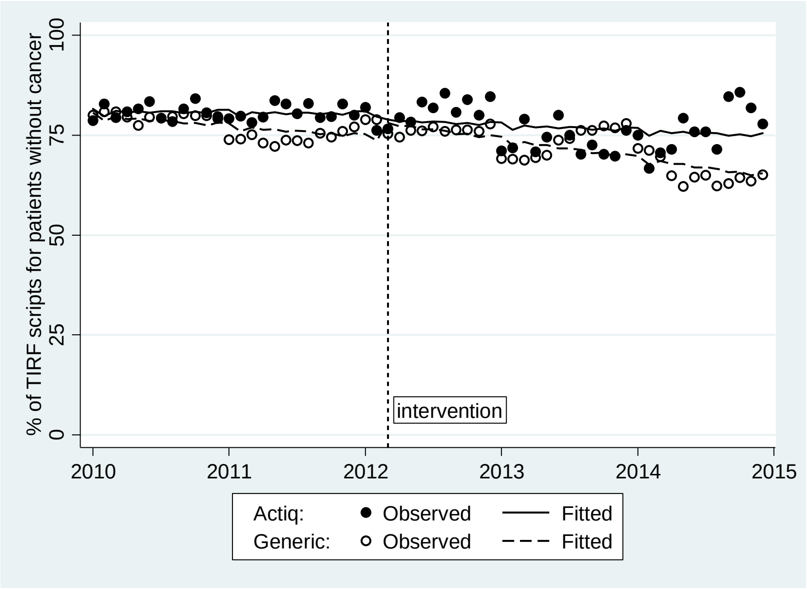


**B**

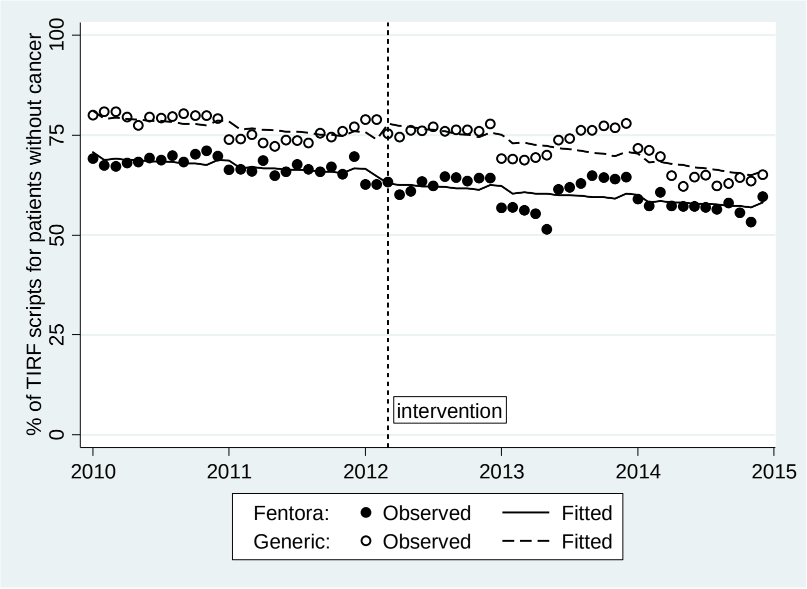

**eFigure 11.** Monthly percentage of transmucosal immediate-release fentanyl (TIRF) prescriptions filled by patients without cancer diagnosis, by TIRF brand. Dashed vertical line denotes TIRF-REMS implementation in March 2012.

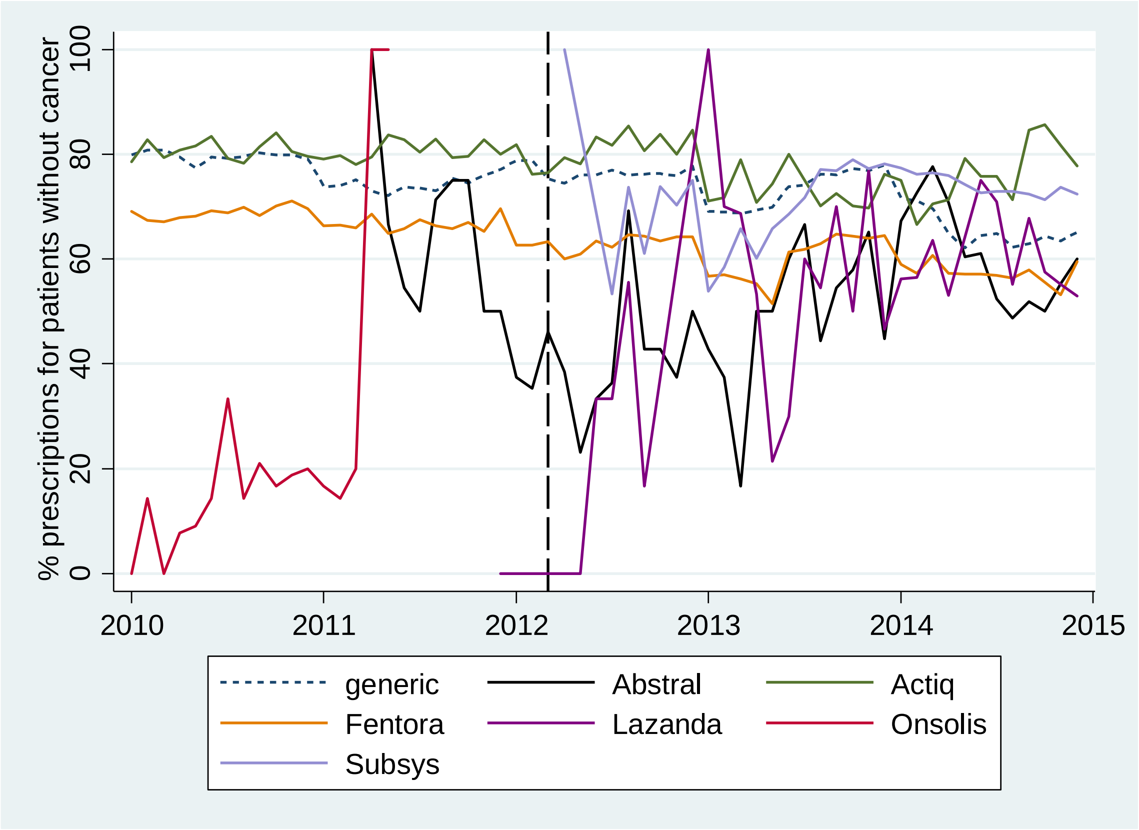

**eFigure 12.** Adjusted interrupted time series model of monthly percentage of transmucosal immediate-release fentanyl prescriptions to patients without known opioid tolerance, 2010-2014. For this analysis we excluded the first 3 months of 2010, since we used an up-to 90-day look-back period to establish patients' prescribing history and opioid tolerance. Dotted line denotes TIRF-REMS implementation in March 2012. Points represent the raw data and lines represent the adjusted, best fit slope.

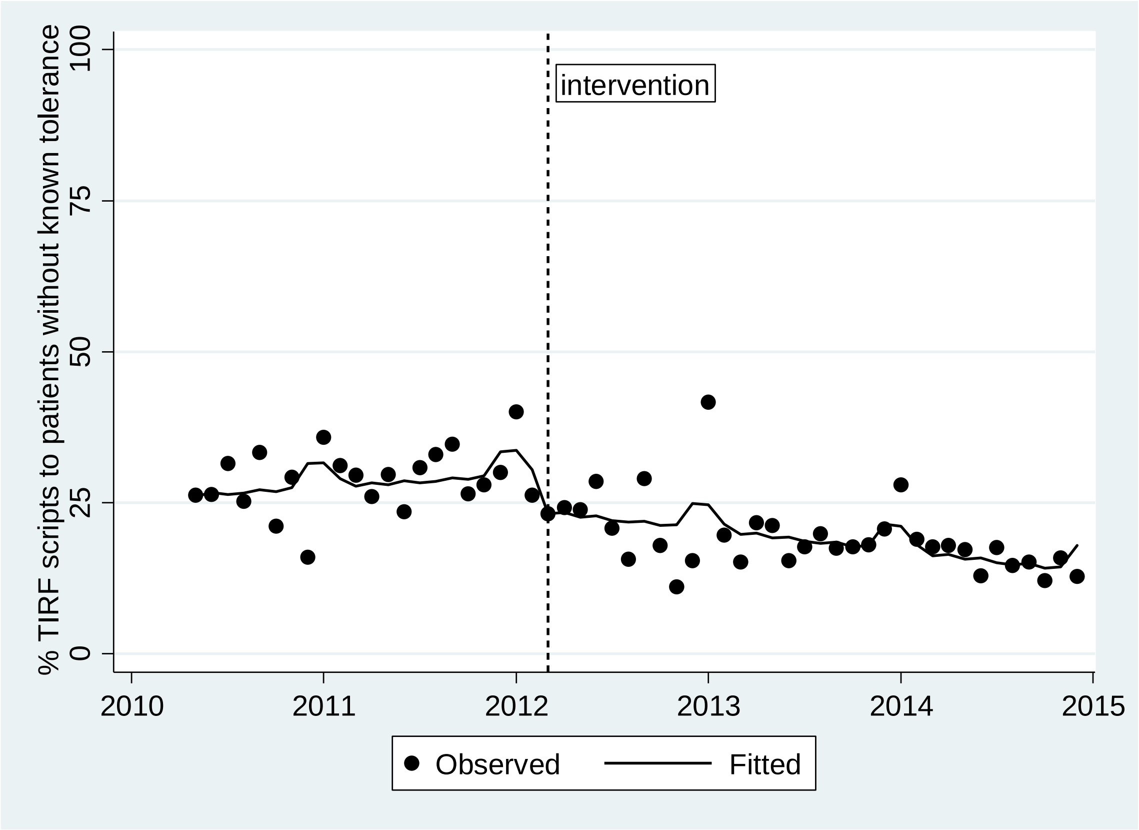

**eFigure 13**. Percent of transmucosal immediate-release fentanyl prescriptions to patients without known opioid tolerance using various lookback periods, 2010-2014. For the study, known opioid tolerance was defined as meeting the tolerance thresholds during any of the lookback periods (red line below). Dotted line denotes month of TIRF-REMS implementation.

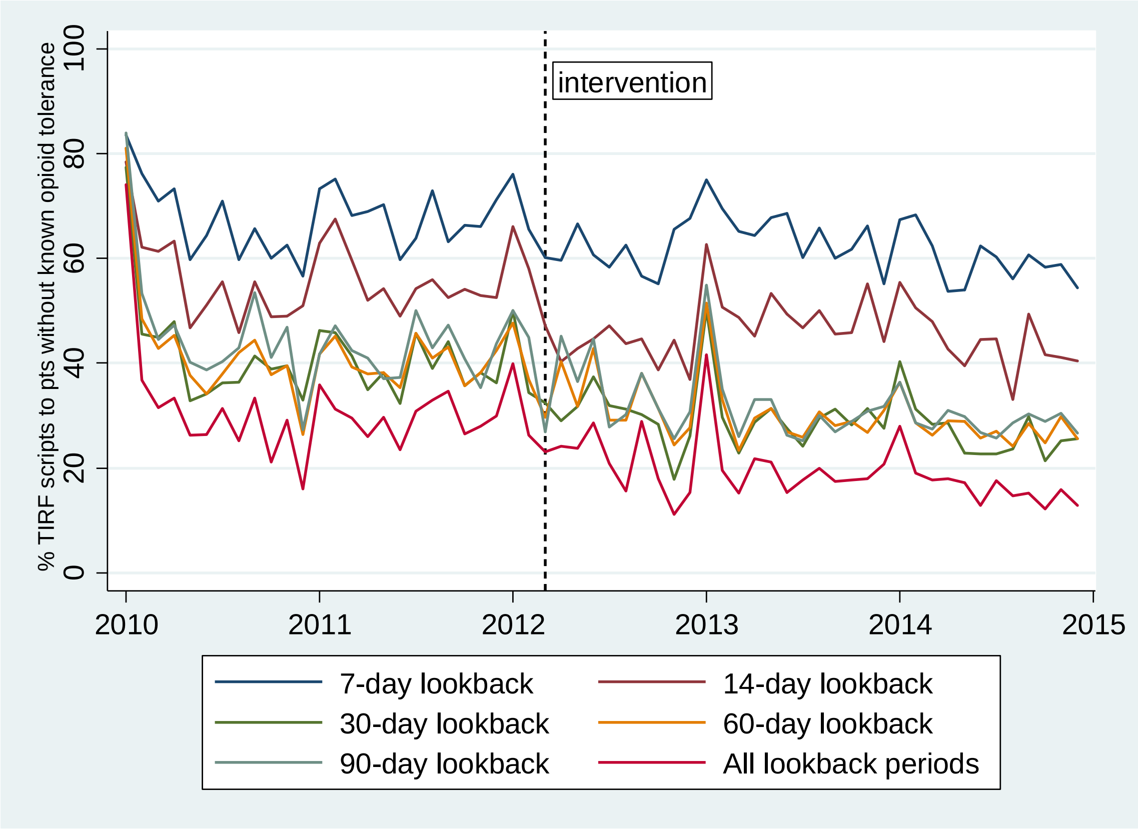

**eFigure 14**. Adjusted interrupted time series model of the monthly percentage of generic vs Fentora-branded transmucosal immediate-release fentanyl prescriptions to patients without opioid tolerance, 2010-2014. Dotted line denotes month of REMS implementation. When compared to generic prescriptions, there were no significant differences in the pre-TIRF-REMS trend among Fentora prescriptions (0.18%, 95% CI, -0.35, 0.39; p=0.92), nor in the level change (7.56%, 95% CI, -0.80, 15.9; p=0.08) or trend change after TIRF-REMS implementation (0.47%, 95% CI, -1.07, 0.12; p=0.12).

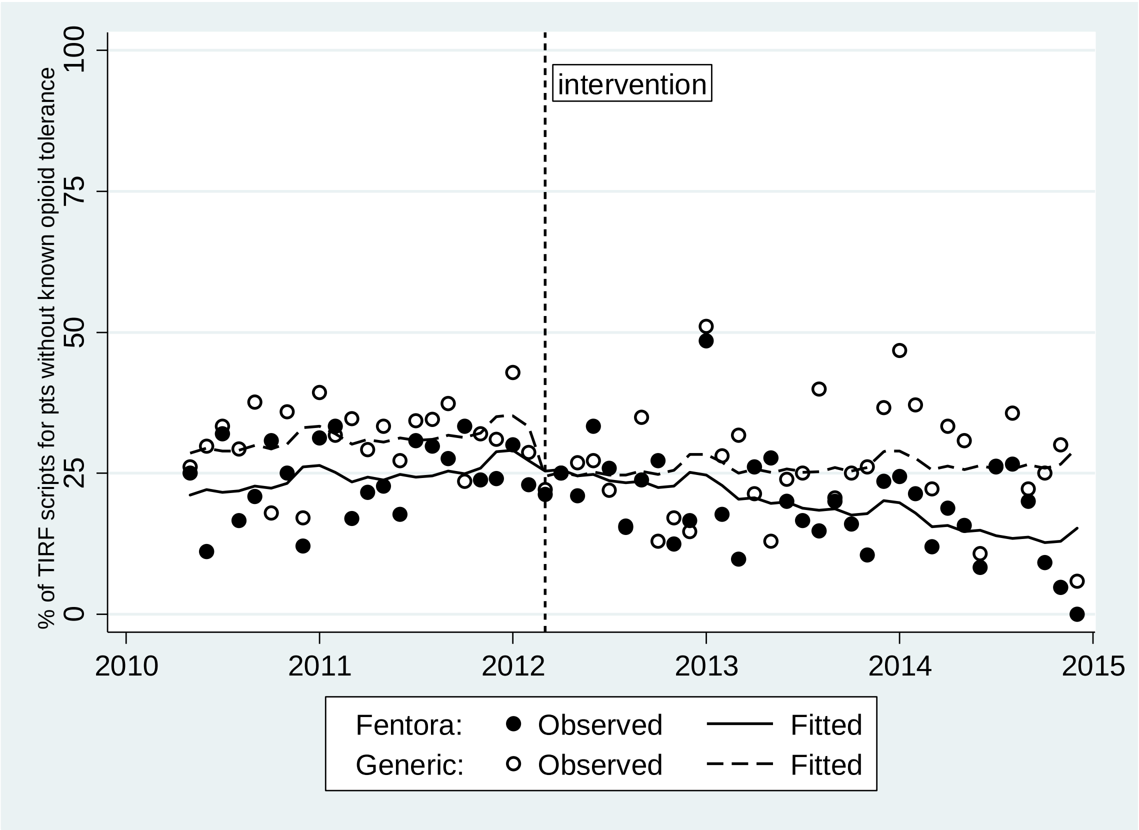

eReferences


1. Wagner AK, Soumerai SB, Zhang F, Ross-Degnan D. Segmented regression analysis of interrupted time series studies in medication use research. J Clin Pharm Ther. 2002;27:299–309.

2. Linden A. Conducting interrupted time-series analysis for single-and multiple-group comparisons. Stata J. 2015;15:480–500.

3. Humphreys DK, Gasparrini A, Wiebe DJ. Evaluating the Impact of Florida's "Stand Your Ground" Self-defense Law on Homicide and Suicide by Firearm: An Interrupted Time Series Study. JAMA Intern Med. 2017;177:44–50.

4. Linden A. A comprehensive set of postestimation measures to enrich interrupted time-series analysis. Stata J. 2017;17:73–88.
